# Prediction and characterisation of lantibiotic structures with molecular modelling and molecular dynamics simulations

**DOI:** 10.1038/s41598-019-42963-8

**Published:** 2019-05-09

**Authors:** Hirak Jyoti Chakraborty, Aditi Gangopadhyay, Abhijit Datta

**Affiliations:** 1 Central Inland Fisheries Research Institute, Barrackpore, Kolkata, 700120 West Bengal India; 2 Department of Chemical Technology, University of Calcutta, 92 APC Road, Kolkata, 700009 West Bengal India; 3 Department of Botany, Jhargram Raj College, 721507 Jhargram, West Bengal India

**Keywords:** Protein structure predictions, Protein structure predictions, Proteome informatics, Proteome informatics

## Abstract

Lantibiotics are lanthionine-containing bactericidal peptides produced by gram-positive bacteria as a defence mechanism against other bacterial species. Lantipeptides disrupt the integrity of target cells by forming pores in their cell membranes, or by preventing cell wall biosynthesis, which subsequently results in cell death. Lantibiotics are of immense importance to the food preservation and pharmaceutical industries. The rise in multidrug resistance demands the discovery of novel antimicrobials, and several authors advocate that lantibiotics hold the future of antimicrobial drug discovery. Owing to their amenability to structural modifications, novel lantibiotics with higher efficacy and antimicrobial activity can be constructed by bioengineering and nanoengineering strategies, and is opined to have immense therapeutic success in combating the rise in multidrug resistance. Understanding the structure and dynamics of lantibiotics is therefore crucial for the development of novel lantipeptides, and this study aimed to study the structural properties and dynamics of 37 lantibiotics using computational strategies. The structures of these 37 lantibiotics were constructed from homology, and their structural stability and compactness were analysed by molecular dynamics simulations. The phylogenetic relationships, physicochemical properties, disordered regions, pockets, intramolecular bonds and interactions, and structural diversity of the 37 lantipeptides were studied. The structures of the 37 lantipeptides constructed herein remained stable throughout simulation. The study revealed that the structural diversity of lantibiotics is not significantly correlated to sequence diversity, and this property could be exploited for designing novel lantipeptides with higher efficacy.

## Introduction

Lantibiotics are ribosomally-synthesised peptide bacteriocins, produced by gram-positive bacteria for targeting other bacterial species during defence strategies, and undergo extensive post-translational modifications prior to forming the mature functional lantipeptide^1,2^. Lantibiotics, or lanthionine-containing antibiotics, are so named because they contain unusual amino acids, lanthionine (Lan) and methyllanthionine (MeLan), which are formed by the fusion of two alanines cross-linked by a thioether linkage^1,3^. Lantipeptides also contain several unsaturated amino acids, including dehydroalanine and dehydrobutyrine^1^.

The bacteriocidal activity of lantibiotics is attributed to the formation of stable pores in the target membrane, which disrupts cellular integrity or prevents cell wall biosynthesis^4–6^. Lantipeptides are highly sought after antimicrobials in the food preservation and pharmaceutical industries owing to their low toxicity in mammalian systems, higher potency than antibiotics, few or no reports of lantibiotic resistance in bacteria, and potent activity against drug-resistant strains such as MRSA and VRE^7–11^. Drug resistance is a serious global concern at present, and the rising emergence of resistant strains demands the design of novel therapeutic strategies. Antibiotic resistant strains often develop biofilms, which further aggravates the crisis of resistance, necessitating the prevention of biofilm formation. The potential of several lantibiotics including nisin, nukacin ISK-1, and gallidermin in hindering the formation of biofilms in staphylococcal strains such as MRSA is widely known^12^. Numerous studies have demonstrated the efficacy of lantibiotics against resistant strains including MRSA, VRE, and GISA^13,14^. Several authors emphasise on the potential of lantibiotics in combating the emerging drug resistant strains and support the view that they can serve as feasible alternatives to antibiotics in the future^15^. Efforts are being made to employ bioengineering strategies for the development of optimised lantipeptides and nano-engineering approaches for broadening the antibacterial spectrum of lantibiotics^16,17^. With the exception of cinnamycin, all the lantibiotics selected herein are lanthionine-containing peptide antibiotics that are able to depolarise the energised bacterial membrane, and subsequently destabilise their membrane integrity. Additionally, the 37 lantipeptides, barring cinnamycin, are capable of creating aqueous transmembrane pores^17^. Although these 36 lantibiotics are functionally similar, their structures are diverse, especially with respect to post-translational modifications, presence of unusual amino acids including dehydrated and unsaturated amino acids with variable linkage patterns, and methyl lanthionine bridges that are crucial to structural stability and function^10,18^. The tertiary structures, structural conformation, important amino acid residues, conserved domains, and intra-molecular chemical bonds need to be understood in further detail for designing engineered lantipeptides with enhanced stability and bioactivity^19^.

In this study we constructed the structures of 37 lantibiotics from over 25 organisms, using molecular modelling approaches, and studied their structural and sequence diversity, in addition to analysing their structural dynamics using molecular dynamics simulations. The lantibiotic sequences selected in this study had reviewed, manually annotated information in UniProtKB, and the existence and function of the 37 lantipeptides were experimentally proven.

## Results

### Sequence-based information

The sequences retrieved from UniProtKB [Supplementary Table S1] belonged to five protein families (InterPro accession IDs: IPR007682, IPR006079, IPR029243, IPR027632, and IPR012519), containing five Pfam detailed signatures (Pfam accession IDs: PF04604, PF02052, PF14867, PF16934, and PF08130). Based on the composition of the conserved domains, the lantibiotics were found to belong to six super families, namely, lantibiotic type A, gallidermin, lantibiotic A, TOMM pelo, mersacidin, and antimicrobial 18. The physico-chemical properties, including the molecular weight, isoelectric point, aliphatic indices, sequence length distribution, extinction coefficients, hydropathy indices, antigenicity, and presence of disordered regions, were determined [Supplementary Figs. S1–S6, Supplementary Table S3].

### Phylogenetic analysis

**Figure 1:**
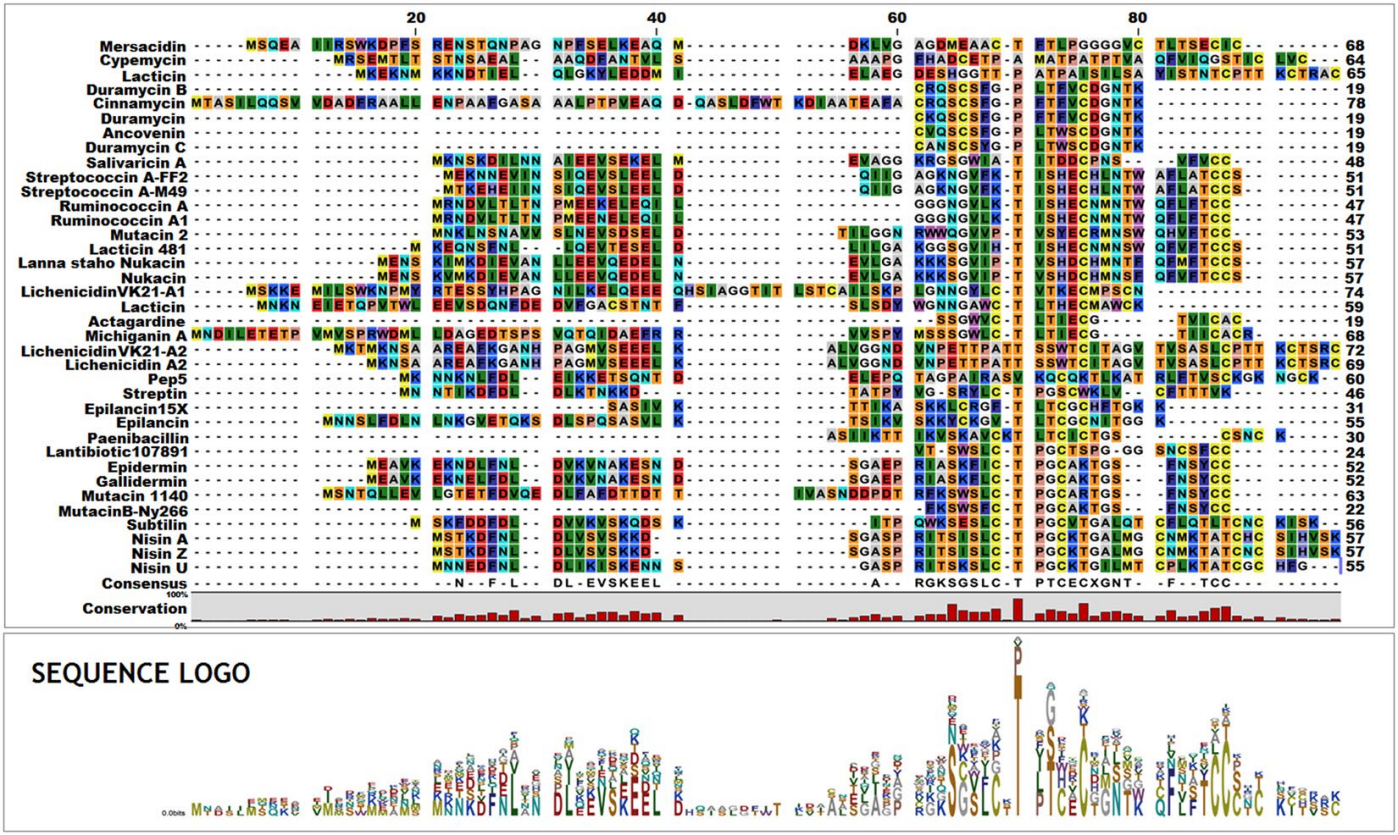
MSA demonstrating the sequence conservedness among the 37 lantipeptides selected for this study. The sequence logo represents the most commonly occurring amino acid at a particular position, where the size of the lettering indicates the frequency of occurrence of a particular amino acid.

**Figure 2:**
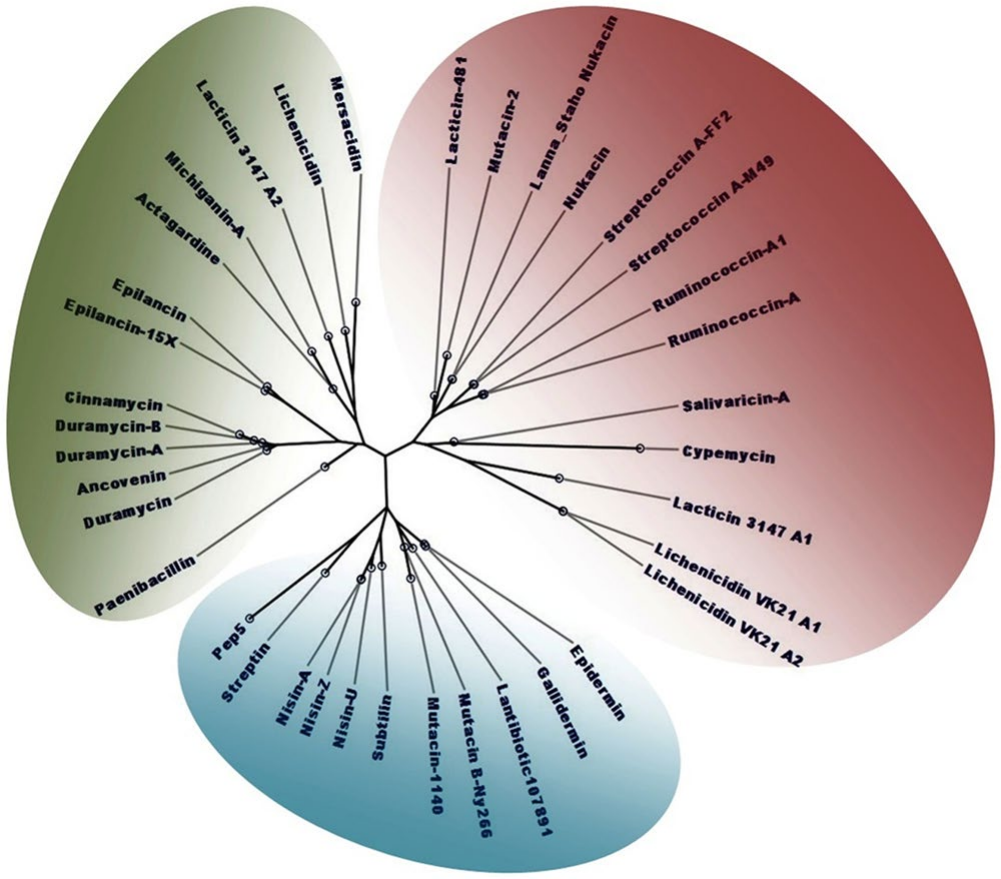
Phylogenetic tree of the 37 lantipeptides, constructed using the Neighbour-Joining algorithm. The 37 lantipeptides were grouped into three groups, which are demarcated by green, blue, and red colours.

The multiple sequence alignment (MSA) revealed that the 37 lantibiotic sequences shared a reasonable degree of sequence similarity [Fig. 1]. The Neighbour-Joining phylogenetic tree demonstrated that the sequences belonged to three distinct evolutionarily-related clusters. The nisins (A, Z, and U) were clustered in the same group as epidermin, gallidermin, mutacins, subtillin, streptin, and pep5 [Fig. 2]. The duramycins and epilancins were grouped along with mersacidin, lacticin, actagardine, cinnamycin, ancoverin, and paenibacillin. The third group comprised the ruminococcins, mutacin2, lichenicidins, salivaricin, streptococcin, nukacins, and cypermicin [Fig. 2]. This third group could be further sub grouped into two - with salivaricin A, cypemycin, lacticin 3147 A1, and the lichenicidins in one subgroup, and lacticidin 481, mutacin 2, the nukacins, streptococcins, and ruminococcins in the other.

### Comparative modelling, validation, and analysis

**Figure 3:**
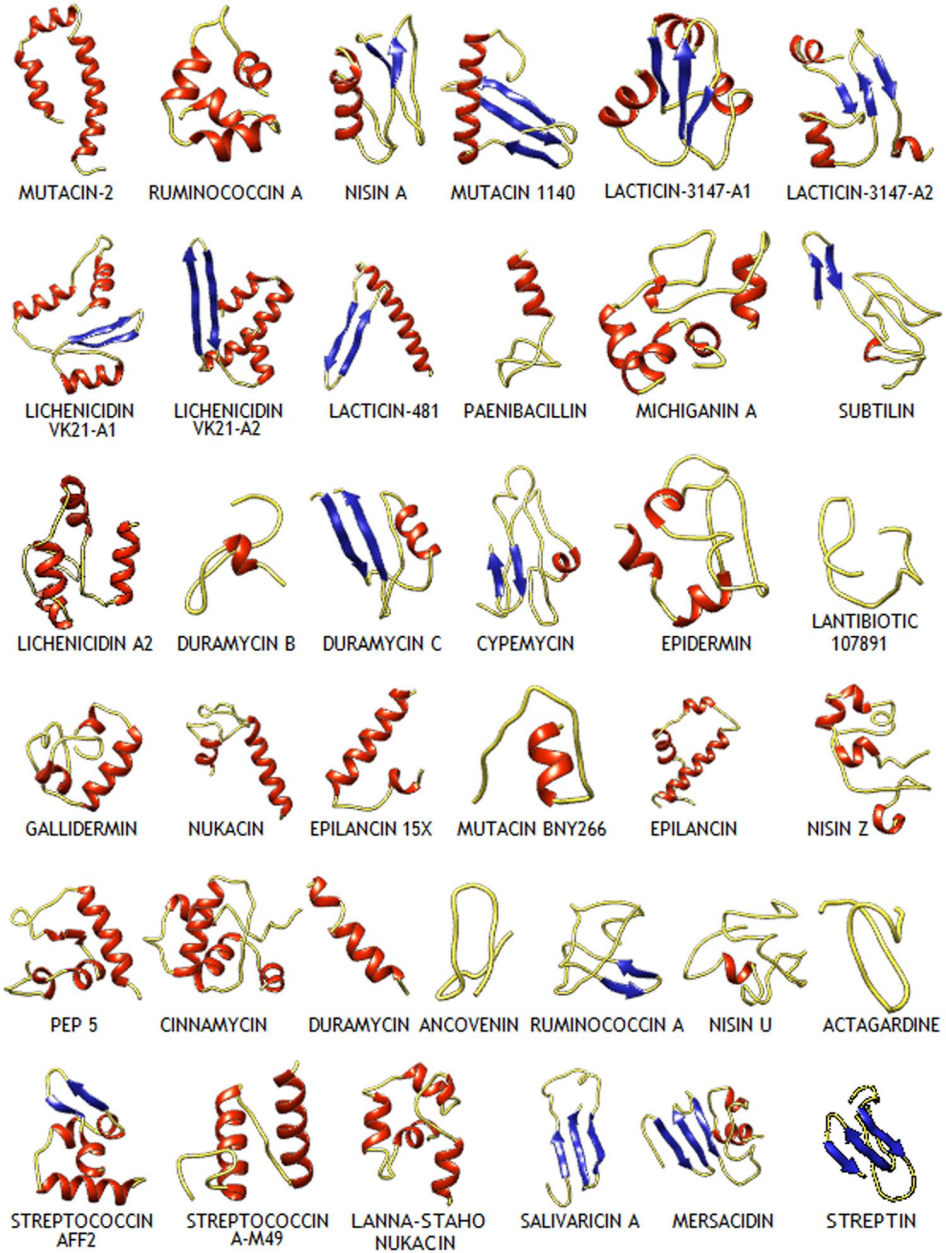
Structures of the 37 lantibiotics constructed by homology modelling in ribbon representation.

The structures of the 37 lantipeptides constructed from homology are represented in Fig. 3. The models were comparable to experimentally-derived protein structures of similar length, as indicated by the ProSA Z-score and the global quality Z-scores obtained from the Verify 3D server. The ProSA Z-scores of the lantibiotic homology models fell within the range of experimentally-derived X-ray and NMR structures of similar length [Supplementary Table S2]. Ramachandran plot analyses indicated the proper assignment of backbone torsion angles, with the torsion angles of the majority of residues being within the allowed regions of the Ramachandran plot [Supplementary Table S2]. Additionally, the different kinds of intermolecular bonds and interactions, including intermolecular hydrogen bonds, van der Waals interactions, disulphide bonds, salt bridges, π-π stacking interactions, and π-cation interactions were determined for each of the 37 lantibiotic models generated herein and subsequently analysed [Supplementary SF1].

### Pockets and disordered residues

Some of the lantibiotics, including lacticin 3147-A1, lacticin 3147-A2, and cypemycin, were found to contain disordered regions that were predicted to have a role in protein binding [Supplementary Table S3]. Additionally, the residues comprising the pockets in the lantibiotic structures were analysed and the details of the pockets and mouths have been tabulated in Table 1.

**Table 1:** Pockets and mouth information of the 37 lantipeptides. Pocket residues that are disordered have been highlighted in grey

Lantibiotic	POCKET INFORMATION				MOUTH INFORMATION				
	ID	Pocket area (sa^*^)	Pocket volume (sa^*^)	Residue composition	Number of mouths	Area_sa^*^	Area_ms^**^	Len_sa^*^	Len_ms^**^
Mutacin-2	1	56.931	32.903	SER11, GLU14, VAL15, LEU20, ILE23, ARG28, TRP29	1	26.154	81.30	35.262	44.06
	2	41.31	20.996	ALA8, SER11, LEU12, TRP29, VAL33, THR36	2	13.341	63.63	27.182	44.77
Ruminococcin-A	1	29.864	7.221	THR9, GLU16, ILE20, TRP40, LEU43, PHE44, CYS46	1	8.57	46.03	22.816	31.61
	2	14.323	4.109	MET1, ASP4, VAL5, LEU8, CYS35, ASN36	2	6.592	33.68	15.353	24.15
Nisin-A	1	34.295	10.211	ASP5, MET44, LYS45, THR46, THR48, CYS49	1	5.878	32.58	15.987	24.78
	2	27.773	8.290	LYS15, LYS16, SER18, GLY19, THR31, PRO32, GLY33, CYS51, ILE53	1	5.848	27.95	13.375	22.17
Mutacin-1140	1	92.325	81.968	MET1, SER2, LEU7, VAL9, PHE22, PHE24, PHE42, TRP45, SER60, TYR61, CYS62	1	50.752	116.08	42.267	51.06
	2	27.22	14.609	VAL17, GLN18, GLU19, LYS43, SER46, LEU47	1	14.326	45.91	19.006	27.80
Lacticin 3147 A1	1	36.210	14.434	TRP13, GLU15, ASN33, PHE35, THR49, LEU50, THR51, CYS54	1	5.556	26.55	12.056	20.85
	2	32.778	11.784	GLU15, GLU16, SER31, THR32, ASN33, GLY45, ALA46, TRP47, CYS48, THR49	1	12.838	52.97	24.581	33.38
Lacticin 3147 A2	1	132.978	81.913	GLN15, LEU16, GLY17, TYR19, MET24, LEU27, GLU29, GLY30, ASP31, SER33, HIS34, THR38, ALA40, THR41, ALA43	3	20.937	80.24	31.399	57.79
	2	56.321	39.172	, ILE12, LEU16, LYS18, ASP23, ILE25, GLU26	1	40.159	116.81	50.794	59.59
Lichenicidin VK21 A1	1	112.316	46.387	SER38, ILE39, ALA40, GLY42, LEU52, SER53, LEU56, ASN58, ASN59, GLY60, TYR61, LEU62, CYS73, ASN74	3	11.664	66.77	27.840	54.23
	2	49.694	8.986	, ILE27, LEU28, LEU31, HIS37, ILE39, ASN59, GLY60, TYR61, ASN74	2	2.314	24.03	8.606	26.20
Lichenicidin VK21 A2	1	95.152	44.844	MET4, SER7, ALA8, GLU11, ASN17, ALA20, GLY21, VAL23, SER24, THR69, CYS72	1	19.996	82.33	40.174	48.97
	2	58.933	22.010	, MET4, THR41, ALA54, GLY55, VAL56, VAL58, SER70, ARG71	2	16.723	66.09	28.280	45.87
Lacticin-481	1	49.526	21.214	GLN11, GLU15, LEU18, ILE31, HIS32, THR33, GLN44, VAL46	1	25.448	91.30	42.822	51.62
	2	4.649	0.584	LEU18, ASP19, LEU22, ILE31, VAL46, THR48	1	0.987	13.93	5.294	14.09
Paenibacillin	1	31.872	16.332	LYS9, ALA13, VAL14, LYS16, CYS20	1	16.909	61.87	29.357	38.15
	2	13.079	1.590	SER11, ALA13, ILE21, CYS22, SER25, CYS26, SER27	2	1.184	25.48	8.556	26.15
Michiganin-A	1	89.285	36.207	ARG16, TRP17, ASP18, MET19, LEU20, VAL43, TYR46, MET47, ILE63, ARG68	1	22.255	68.16	29.017	37.81
	2	15.530	2.124	ALA23, GLY24, ASP26, THR27, GLN32, GLN34	1	2.756	21.18	8.765	17.56
Subtilin	1	9.903	1.615	THR22, GLN24, SER29, LEU30	1	2.306	25.08	13.663	22.46
Lichenicidin A2	1	250.794	128.956	PHE10, HIS15, PRO16, ALA17, GLY18, MET19, VAL20, SER21, GLU24, LEU25, LEU28, ASN35, THR38, THR39, THR42, THR43, TRP46, GLY52, VAL55, SER56, CYS60, PRO61, THR62, THR63, LYS64, CYS65, THR66	1	30.200	84.29	35.034	43.83
	2	44.818	35.937	, ALA6, MET19, VAL20, LEU25, VAL34, THR39, PRO40, THR43	1	25.906	70.12	27.564	36.36
Duramycin B	1	11.144	1.013	CYS5, SER6, PHE7, THR11, VAL13, CYS14, ASN17, THR18	1	0.010	4.88	0.643	9.44
	2	1.237	0.128	ARG2, CYS14, ASP15, THR18	1	0.516	11.60	3.521	12.32
Duramycin C	1	20.801	9.875	ASP14, VAL15, LYS16, ALA42, LYS43	1	9.296	40.65	18.016	26.81
Cypemycin	2	46.591	7.798	, ALA15, LEU16, ALA17, VAL25, LEU26, ALA41, MET42, PHE53	2	0.667	21.8	7.078	24.67
Epidermin	1	37.588	9.316	LYS20, PHE35, ILE36, CYS41, THR44, GLY45, PHE47, ASN48	2	4.271	36.47	18.179	35.77
	2	15.318	4.025	MET1, ASP9, LEU10, LEU13, CYS51	1	2.942	24.57	11.533	20.33
Lantibiotic 107891	1	6.857	1.098	VAL1, TRP4, SER13, SER18, ASN19, CYS20	1	0.616	19.25	8.915	17.71
	2	1.145	0.094	VAL1, THR2, SER3, CYS7	1	0.398	10.11	3.176	11.97
Gallidermin	1	9.957	1.250	PHE11, ASP14, VAL15, ASN18, PHE35, THR38, TYR50, CYS51	1	0.561	11.29	3.902	12.70
	2	8.522	0.943	ASN23, ASP24, SER25, GLY26, LEU36, CYS37	1	2.261	18.57	7.726	16.52
Nukacin	1	20.934	15.189	GLU11, VAL12, LEU15, SER34, GLY35, VAL36	1	35.768	82.48	29.092	37.89
	2	2.615	0.077	GLU18, VAL19, LYS31, LYS32, GLY35, VAL36	2	0.025	11.00	1.089	18.68
Epilancin 15×	1	77.601	60.827	LYS13, CYS16, ARG17, LEU21, THR22, CYS23, CYS25, PHE27	1	30.833	79.91	31.011	39.81
	2	25.339	19.239	VAL5, ILE9, HIS26, PHE27, LYS30,	1	20.993	56.99	21.315	30.11
Mutacin B-NY266	1	28.755	5.461	LYS2, SER3, PHE6, CYS11, ALA12, PHE17, ASN18, SER19	1	1.110	15.46	6.031	14.83
	2	13.174	1.588	SER3, PHE6, CYS7, SER19, TYR20, CYS21	2	0.361	15.45	5.492	23.09
Epilancin	1	215.553	113.859	LEU10, GLY13, VAL14, GLN17, LYS18, LEU21, LEU29, LYS30, ILE33, VAL35, TYR39, CYS40, VAL43, THR44, THR46, CYS47, GLY48, CYS49	2	41.176	157.24	75.004	92.60
	2	106.021	88.529	LEU5, PHE6, ASN9, LEU10, ILE51, THR52, GLY53, GLY54, LYS55	1	49.206	136.48	57.939	66.74
Nisin-Z	1	16.422	14.287	CYS30, THR31, CYS51, HIS54, SER56, LYS57	1	26.449	67.13	29.058	29.06
	2	0.928	0.092	LEU10, LYS15, PRO22, ILE53	1	0.470	10.72	3.157	11.95
Pep5	1	97.688	25.747	LEU23, GLN26, THR27, ALA28, PRO30, ALA31, LEU43, LYS44, ALA45, THR46, ARG47, LEU48, GLY58, CYS59, LYS60	2	6.879	45.01	19.609	37.20
	2	37.560	11.377	GLU11, LYS14, GLU15, ASN19, THR20, GLU22, ALA28, GLY29	1	5.195	25.32	10.695	19.49
Streptococcin A-FF22	1	54.147	34.349	ILE5, ALA18, GLU21, ASN22, ALA24, PHE26	1	23.116	65.29	26.667	35.46
	2	126.691	31.250	ALA28, SER29, ALA30, ALA31, ALA32, LEU33, VAL37, GLU38, ASP41, GLN42, SER44, LEU45, SER65, PHE66, PRO68, PHE69, PHE71	2	0.653	22.13	6.605	24.20
Lanna-Staho nukacin	—	—	—	—	—	—	—	—	—
Nisin-U	1	19.454	5.690	CYS1, LEU10, CYS14, GLY16	2	4.366	34.92	14.445	32.04
	2	14.993	2.331	CYS1, VAL2, GLN3, CYS5, GLY8, LEU10, GLY16, ASN17	1	0.410	10.85	3.198	11.99
Streptococcin A-M49	1	30.268	8.081	GLU14, GLU16, GLN19, SER32, HIS33, ASN36	2	2.976	29.03	11.703	29.30
	2	2.827	0.583	ASN3, GLU14, HIS33, GLU34	1	2.546	19.09	9.277	18.07
Salivaricin-A	1	119.605	69.104	GLU4, ASP5, PHE6, ILE13, LYS15, ASN18, SER19, GLY20, ALA21, SER22, LYS28, SER29, LEU30, CYS31, THR32	1	13.804	69.36	35.499	44.30
	2	103.728	36.243	MET1, ASN2, ASN3, PHE6, LEU8, ILE25, THR26,SER29, LEU30, THR42, LEU45, PHE54, GLY55	2	5.063	41.41	20.920	38.51
Duramycin	1	4.072	0.602	LEU8, GLU11, CYS12, GLY13, ILE16, CYS17	1	0.483	11.10	3.433	12.23
	2	0.232	0.023	SER1, TRP4, THR14	1	0.292	10.83	3.129	11.92
Cinnamycin	2	10.283	1.704	, VAL7, SER10, PHE31, LEU46, CYS50	1	0.389	9.41	3.338	12.13
Ruminococcin-A	1	64.070	37.328	LYS3, GLU4, HIS5, GLU6, ASN9, SER10, GLU13, VAL14, GLU17, GLU18, GLN21	1	27.846	90.92	40.943	49.74
	2	40.389	23.274	GLN12, LEU16, LEU19, PHE31, SER35, HIS36, HIS39	1	16.667	58.29	25.761	34.56
Mersacidin	1	73.437	34.794	, ILE6, MET7, CYS44, HIS45, MET46, ASN47, PHE49, MET52, PHE53	2	32.166	98.93	38.889	56.48
	2	23.867	4.731	ILE10, GLU23, LEU24, VAL27, PHE49, PHE51, MET52, PHE55	1	2.040	17.49	8.811	17.61
Streptin	1	55.864	27.016	GLU22, VAL23, GLY26, LYS27, ARG28, SER30,TRP32, CYS48	1	19.591	72.79	33.854	42.65
	2	7.240	1.899	GLU13, PHE45, CYS47	1	4.864	26.16	11.106	19.90
Actagardine	1	38.730	9.043	PHE14, ARG16, SER29, GLU30, LEU31, VAL59, LEU62, THR63, CYS66, ILE67	1	0.004	6.34	0.312	9.11
	2	29.320	7.472	LEU39, GLY43, ASP44, ALA48, PHE51,GLU65, CYS66, CYS68	1	3.367	25.72	11.950	20.75
Ancovenin	1	59.872	12.390	LYS13, ASN15, LYS16, LYS17, ASP18, THR19, GLY33, SER34, CYS35, LYS46	2	1.336	21.82	8.462	26.05
	2	18.786	5.716	THR19, THR21, SER34, VAL45, LYS46	1	3.493	22.49	9.838	18.63

^*^sa: solvent accessible, ^**^ms: molecular surface.

### Structural diversity of lantibiotics

**Figure 4:**
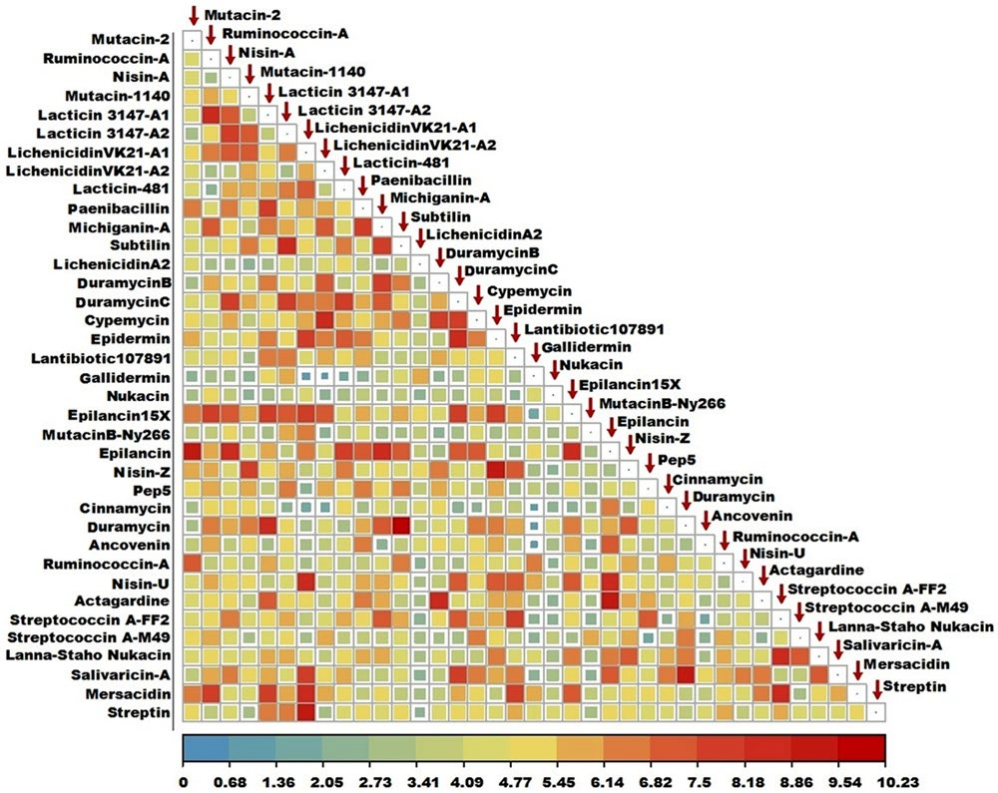
Plot of the structural RMSD, demonstrating the range of structural divergence among the 37 lantibiotics. The colour key provides the range of the structural RMSD (in Å), ranging from a low structural RMSD (blue-green), medium (yellow-orange), to high structural RMSD (red).

**Figure 5:**
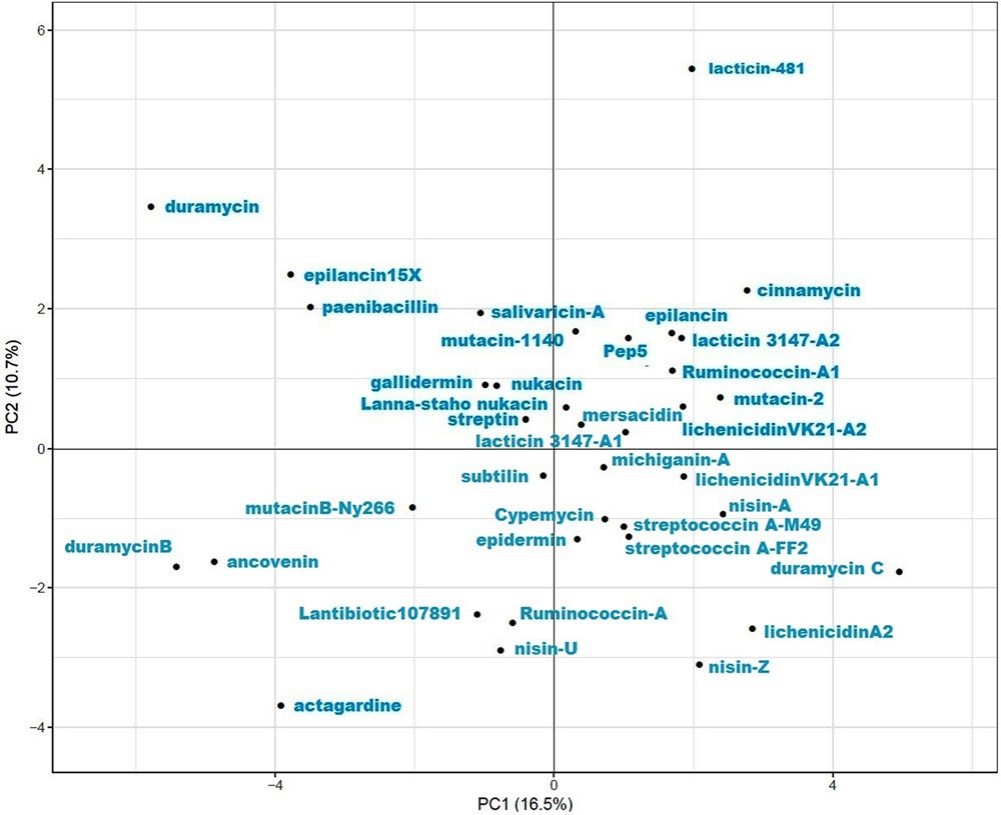
Plot showing the PCA of the 37 lantipeptides with respect to their intra-RMSD values, where the X and Y axes depict principal component 1 (PC1) and principal component 2 (PC2).

The structural diversity of the 37 lantibiotics was reflected in the RMSD values, which in some cases were as high as 10 Å, as represented in Fig. 4. The structural RMSD values of gallidermin with lichenicidin VK21-A2, ancovenin, and cinnamycin were the lowest, being 0.753 Å, 0.837 Å, and 0.934 Å, respectively. The structures of subtilin and duramycin demonstrated the greatest structural diversity, with an RMSD value of 10.226 Å between the two structures. On an average, the structural RMSD values were in the range of 4–5 Å. The average RMSD of galliderim, nukacin, and mutacin B-Ny266 with all the other lantibiotics were the lowest, being in the range of 3–3.5 Å. The relational RMSD data matrix [Supplementary SF2] of all the 37 lantibiotics were standardised prior to the Principal Component Analysis (PCA). The X and Y axis depicted principal component 1 (PC1) and principal component 2 (PC2) respectively, which represented 16.5% and 10.7% of the total variance Fig. 5. The variance explained by the principal components, the value of the principal components, and the value of component loading are provided in the supplementary [Supplementary SF3]. Analysis of the PCA plot revealed that duramycin, duramycin B, duramycin C, lacticin-481, actagardine, and ancovenin had the maximum variation among all the 37 lantibiotics.

**Figure 6:**
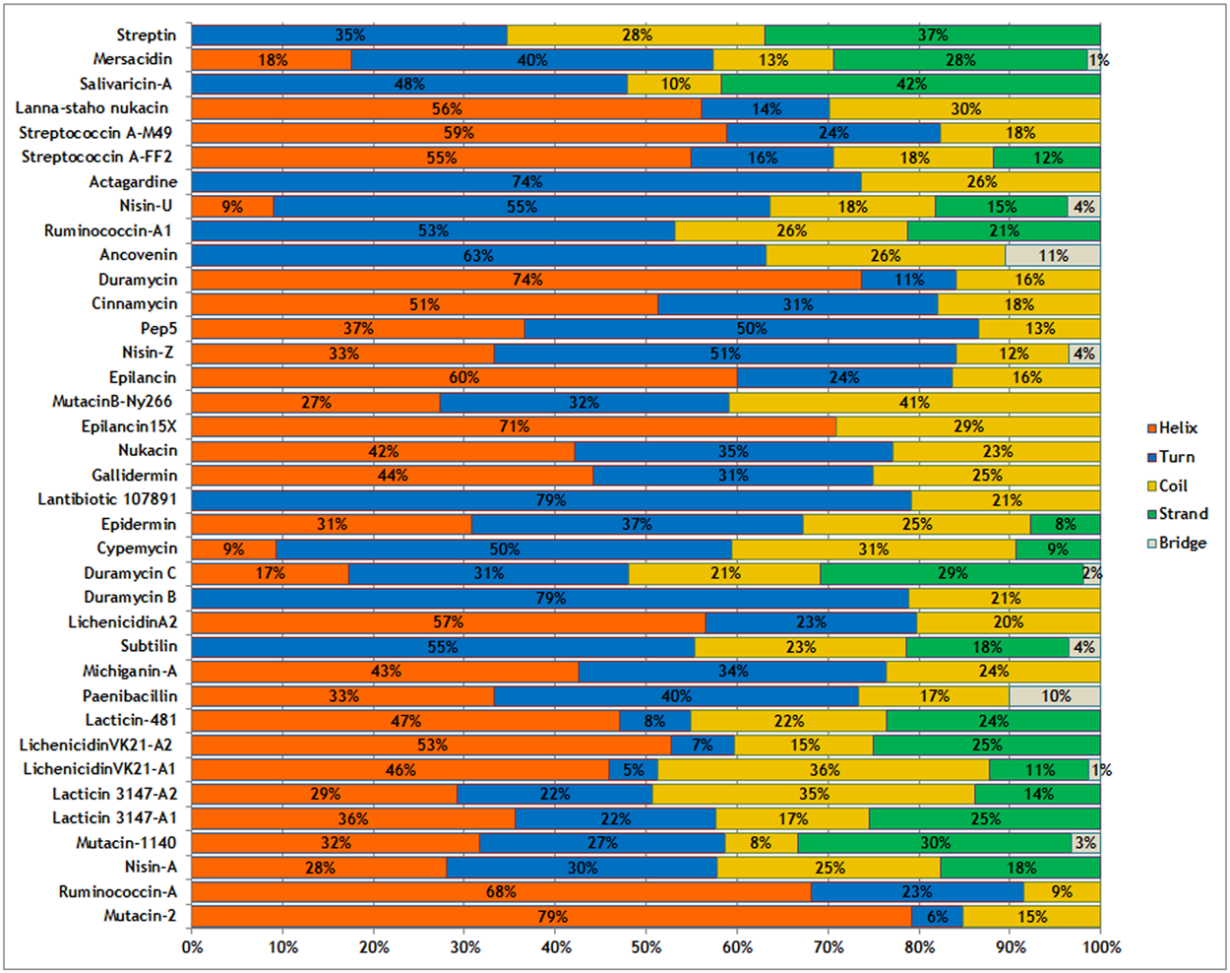
Graphical representation of the secondary structure content of the 37 lantipeptides.

The secondary structure composition of the lantipeptides also varied, with some lantipeptides, including mutacin-2, ruminococcin-A, lichenicidin VK21-A1, lichenicidin VK21-A2, lacticin-481, gallidermin, nukacin, epilancin-15X, epilancin, cinnamycin, duramycin, strepcoccin A-FF2, streptococcin A-M49, and lanna-staho nukacin having a higher helical content [Fig. 6]. On the other hand, mersacidin, salivaricin A, actagardine, nisin U, ruminococcin A1, ancovenin, pep-5, nisin Z, mutacin B-Ny266, lantibiotic 107891, epidermin, cypemycin, duramycin C, duramycin B, and subtilin had a higher content of turns and coils. Among the 37 lantipeptides, the beta-strands were prominent in the structures of streptin, mersacidin, salivaricin A, duramycin C, lacticin 481, lichenicidin VK21-A1, lacticin 3147-A1, and mutacin 1140.

### MD simulation

**Figure 7:**
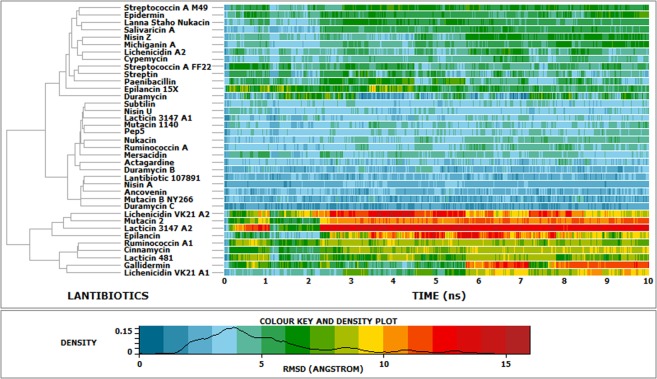
Plot showing the backbone RMSD of the 37 lantibiotics over the simulation time.

**Figure 8:**
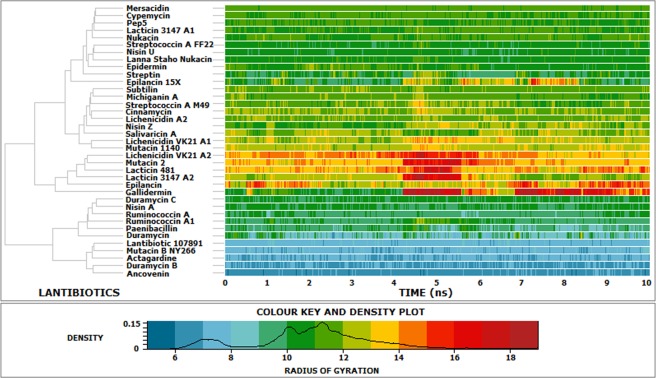
Plot showing the radius of gyration (RoG) of the 37 lantibiotics throughout the simulation time.

**Figure 9:**
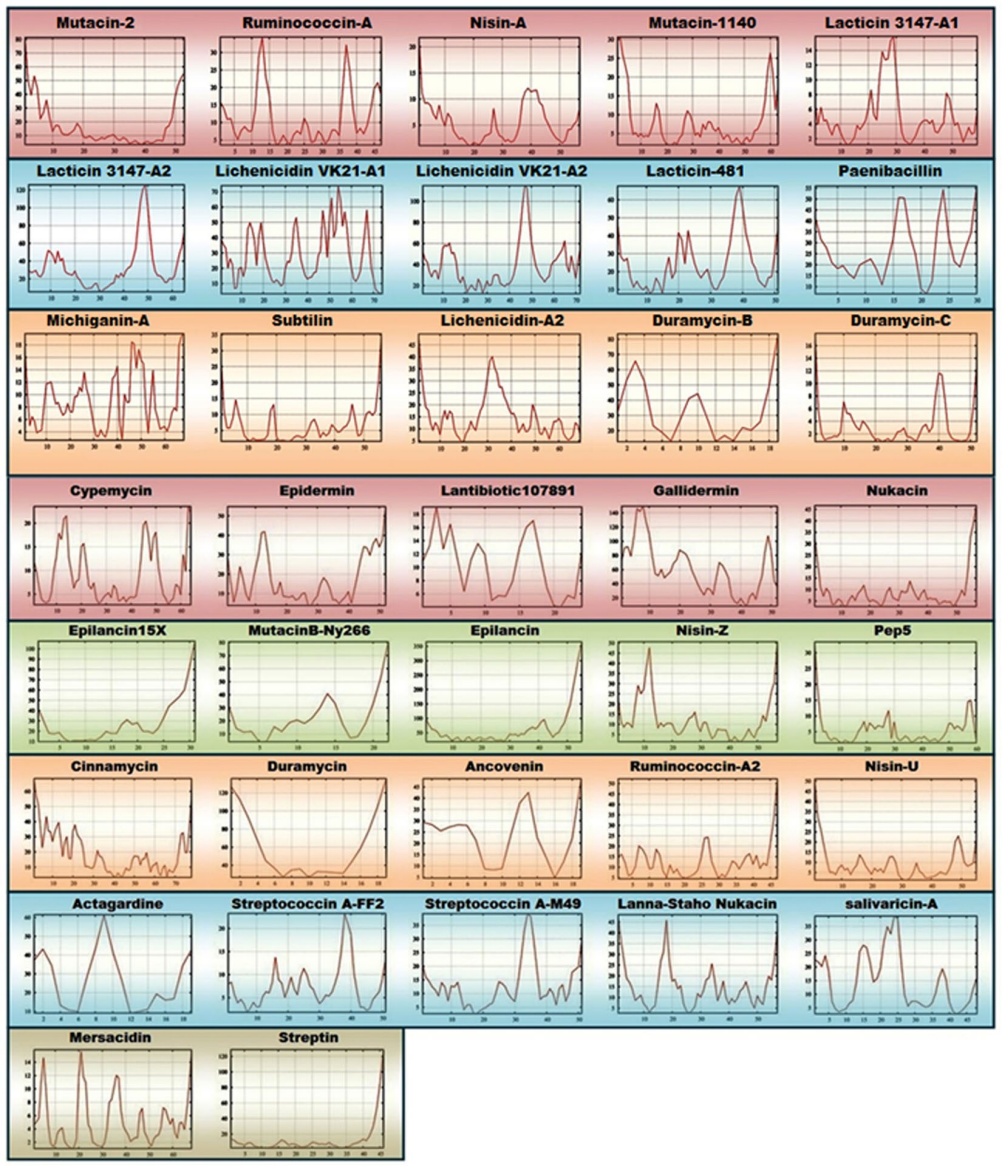
RMSF plots demonstrating the residual fluctuations of the 37 lantipeptides, indicating the flexible regions.

The lantipeptides demonstrated structural consistency throughout the simulation, indicated by the RMSD and radius of gyration^20^ [Figs. 7 and 8]. The lantipeptides with a higher content of turns and coils, including ancovenin, duramycin B, actagardine, mutacin B-Ny266, and lantibiotic 107891, had the lowest radii of gyration among the 37 lantipeptides. Since the radius of gyration is a measure of structural compactness, it can be said that the structures of ancovenin, duramyin B, actagardine, mutacin B-Ny266, and lantibiotic 107891 were the most compact, while the structures of gallidermin, epilancin, lacticin 3147-A2, lacticin 481, mutacin 2, and lichenicidin VK21-A2 were the least compact among the 37 lantipeptides [Fig. 8 and Supplementary Fig. S7]. The RMSF of the peptide backbone was used to determine the most flexible region of the peptide backbone [Fig. 9]. It was noted that while the backbone RMSDs of most of the lantibiotics remained consistent throughout the simulation, the backbone RMSDs of lichenicidin VK21-A2, mutacin 2, lacticin 3147-A2, epilancin, gallidermin, and lichenicidin VK21-A1 were higher than the rest [Fig. 7 and Supplementary Fig. S8]. Analyses of cluster density, cluster size, and average cluster RMSD revealed that the representative structure from cluster 1 was the best conformation in each case. The representative structures were superimposed with the cluster members to compute the relation between the average RMSD and the global distance test (GDT_TS) [Supplementary Fig. S9].

## Discussion

Lantibiotics are bacteroicidal peptides characterised by the presence of unusual amino acids - the thioether-containing polycyclic lanthionines and unsaturated amino acids^1^. They are produced by gram-positive bacteria for targeting other bacterial species by forming pores in the target membrane that disrupt cellular integrity or inhibit cell wall biosynthesis^9^. Lantibiotics are widely used in the food preservation and pharmaceutical industries^7^. In the present global scenario, the surge in the development of drug-resistant strains demands the development of novel drugs and antimicrobials for combating the emerging drug resistance. The high *in vitro* potency combined with the variety of strategies employed for effectively targeting bacterial cells, makes lantibiotics a promising macromolecule for the generation of novel antibiotics in the future^15,21,22^. Lantibiotics inspire the construction of engineered antimicrobial peptides for combating specific bacterial diseases, making the understanding of lantibiotic structures a necessary and important one^7,17^. The objectives of this study were to construct the structures of 37 lantipeptides having reviewed and annotated sequence information in UniProtKB using homology modelling, and to evaluate the diversity, compactness, and stability of the structures of the 37 lantipeptides.

Analysis of the MSA revealed that the lantibiotic sequences shared a high degree of conservedness, which was in marked contrast to the diversity of their structures. The structural diversity of the 37 lantipeptides was determined from the RMSD values. The correlation coefficient between the sequence diversity and structural diversity of the 37 lantipeptides was 0.189. A value of 0.189 indicated that the structural diversity of the 37 lantibiotics is not significantly correlated to the diversity of lantibiotic sequences. This further indicates that the sequence-structure relationship of the lantibiotics selected herein is flexible, allowing room not only for human tailoring, but also explains that the natural post-transcriptional engineering is probably not an accident. Lacticin 3147-A1, lacticin 3147-A2, and cypemycin were found to contain disordered residues that are capable of binding proteins, and some of the residues were also found to comprise the pockets in the lantipeptide structures. Protein-protein interactions involving a disordered protein are generally mediated by a transition from disorder to order upon protein binding^23^. Since protein-protein interactions are often mediated by small flexible pockets at the protein-protein interface, these disordered residues might be responsible for lantibiotic-protein interactions, and could undergo similar structural transitions upon binding.

## Methods

### Lantibiotic sequences

The existence and biological functions of the 37 lantibiotics selected in this study have been established by experimental studies, and the sequences had reviewed and manually annotated information in UniProtKB/Swiss-Prot non-redundant sequence database^24^ [Supplementary Table S1].

### Information from primary data

The domains, repeats, super families, and conserved patterns of the 37 lantibiotics were identified using InterPro Scan and the batch CD-search tool^25,26^. The transmembrane regions and the hydropathy indices of the lantibiotics were determined using the CLC Genomics Work Bench v 8.5. The Kyte-Doolittle and the Eisenberg scales were used for determining the local hydropathy plots. Lantibiotic antigenicity was analysed by the semi-empirical method of Kolaskar and Tongaonkarhas. Information pertaining to the physico-chemical properties, such as molecular weight, isoelectric pH, aliphatic index, hydrophobicity, hydrophilicity, and amino acid composition was also computed. The disordered regions were identified with the DISOPRED3 algorithm^27^.

### Phylogenetic analyses

An MSA of the 37 lantibiotic sequences was generated using the MUSCLE algorithm. The phylogenetic tree was constructed using the Neighbour-Joining algorithm, keeping the bootstrap value at 1000. The CLC Genomics Work Bench v 8.5 was used for phylogenetic analyses.

### Homology modelling, validation, and analysis

The complete structures of the 37 lantipeptides were constructed by homology modelling, using Modeller v 9.11^28,29^. A structure BLAST was performed against the Protein Data Bank (PDB) to identify templates for comparative modelling^30,31^. Template identification was also achieved by the threading-based fold recognition method employed by the PSIPRED server (http://bioinf.cs.ucl.ac.uk/psipred/)^32^. The backbone torsions of the validated models were assessed by analysing their Ramachandran plots, while the improper geometries and clashes were evaluated by checking their stereochemistry, using ProCheck^33^. The quality of the constructed models was additionally estimated by using different servers, including the ProSA II, Verify3D, and PSVS servers^34–36^. The intermolecular bonds and interactions of the 37 structures generated herein were determined using the RING-2.0 web server (http://protein.bio.unipd.it/ring/)^37^.

### Identification of pockets and determination of structural diversity

The secondary structure composition of the lantipeptides were determined with STRIDE (http://webclu.bio.wzw.tum.de/cgi-bin/stride/stridecgi.py)^38^. The pockets were identified using CASTp (http://sts.bioe.uic.edu/castp/), with a probe of radius 1.4 Å^39^. The structural diversity of the lantipeptides was analysed by calculating the RMSD values following structural superimposition of the 37 lantibiotic structures. Each lantipeptide structure was individually superimposed and the intra-RMSD value was computed using CLC Genomics Work Bench v 8.5. In order to understand the structural correlation among the 37 lantipeptides with respect to their intra-RMSD values, a data matrix [Supplementary SF2] of all the 37 lantibiotics were prepared and standardised prior to the PCA. The PCA was performed with the ClustVis tool^40^, where vector scaling is applied to the rows and SVD with imputation is used to calculate the principal components of N = 37 data points.

### Molecular dynamics simulation and trajectory analyses

The structural stability, compactness, backbone flexibility, and per-residue fluctuations were characterised by performing coarse-grained molecular dynamics (MD) simulations of the lantibiotic structures in explicit water. The simulations were performed by combining the four most widely used force fields, namely, Amber, Gromos, OPLS, and CHARMM, in the CABS simulation procedure, run on a high-performance computing server (http://biocomp.chem.uw.edu.pl/CABSflex/)^41,42^. The CABS protein representation was reduced up to four pseudo-atoms per residue, and the sampling was realised by the Monte Carlo method^43^. The simulation length was optimised to obtain the best possible convergence within 10 ns. The trajectories were analysed with VMD and VEGA ZZ^44^. The mean-square-fluctuation [(ΔR)^2^] was calculated using the following equation:

$$\langle {({\rm{\Delta }}R)}_{i}^{2}\rangle =\frac{1}{N}{\sum }_{j}^{N}{(xi(j)-\langle xi\rangle )}^{2}$$

where < > denotes the average across the entire trajectory, *x* represents the position of a particle ***i*** in the frame ***j***, and *N* represents the total number of frames in the trajectory^41,44^.

The trajectories were clustered using the k-means clustering method in such a way that structurally closer models belonged to the same cluster. The best conformation of each lantibiotic was selected after screening the trajectories. Each cluster was superimposed for identifying the best conformation using the Theseus application. The RMSD and RoG of the lantipeptides were determined across the simulation time frame. The root mean square fluctuation (RMSF) was determined for estimating the residual fluctuations, and the most flexible regions were identifiedfrom the RMSF graphs. The stability of the system and the fluctuations across the trajectories were analysed with XMGRACE^45^.

## Supplementary information

Supplementary information

Supplementary SF1

Supplementary SF2

Supplementary SF3
