# Supplementary material for: Prediction and characterisation of lantibiotic structures with molecular modelling and molecular dynamics simulations
Source: Sci Rep. 2019 May 9;9:7169. doi: 10.1038/s41598-019-42963-8 (PMC6509333; doi:10.1038/s41598-019-42963-8)
Supplement: Supplementary file 1 — Supplementary information [file 41598_2019_42963_MOESM1_ESM.doc]

**Prediction and characterisation of lantibiotic structures with molecular modelling and molecular dynamics simulations**

Hirak Jyoti Chakraborty^1^*, Aditi Gangopadhyay^2^, Abhijit Datta^3^

^1^ Central Inland Fisheries Research Institute, Barrackpore, Kolkata 700120, West Bengal, India

^2^ University of Calcutta, 92, APC Road, Kolkata 700009, West Bengal, India

^3^Jhargram Raj College, Jhargram 721507, Paschim Medinipur, West Bengal, India

* Author to whom correspondence should be addressed: Hirak Jyoti Chakraborty, Central Inland Fisheries Research Institute, Barrackpore, Kolkata 700120, West Bengal, India; email address: [hj.chakraborty@gmail.com](mailto:hj.chakraborty@gmail.com)

**
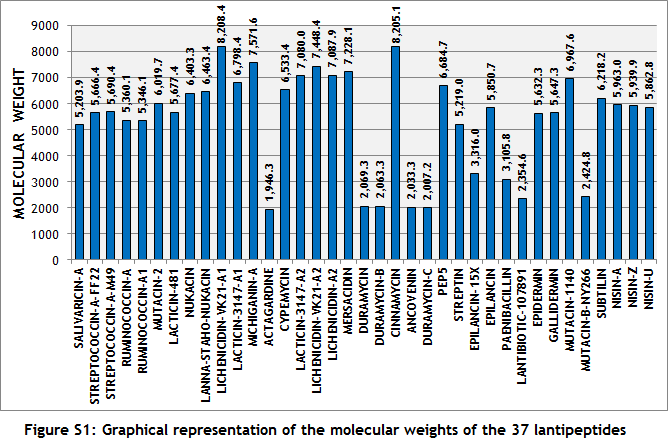
**

**Figure S1: Graphical representation of the molecular weights of the 37 lantipeptide**

**
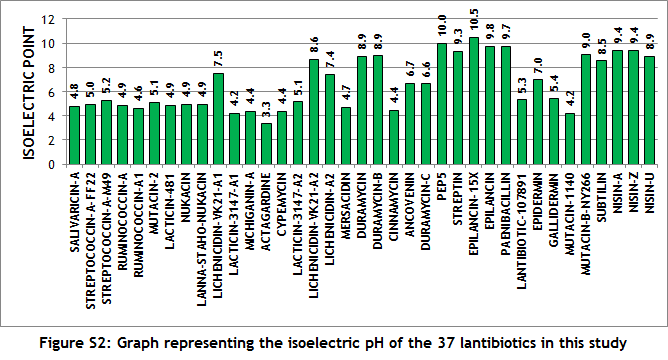
**

**Figure S2: Graph representing the isoelectric pH of the 37 lantibiotics in this study**

**
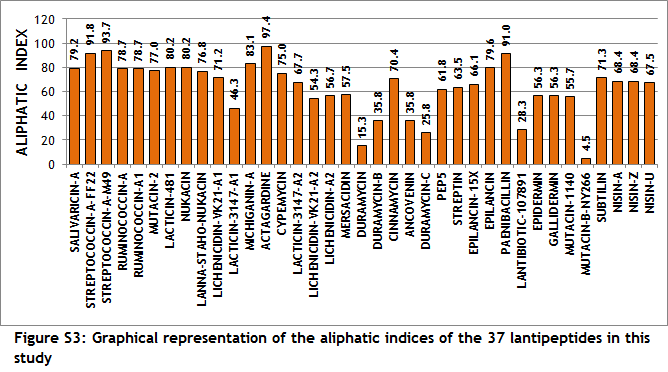
**

**Figure S3: Graphical representation of the aliphatic indices the 37 lantibiotics in this study**

**
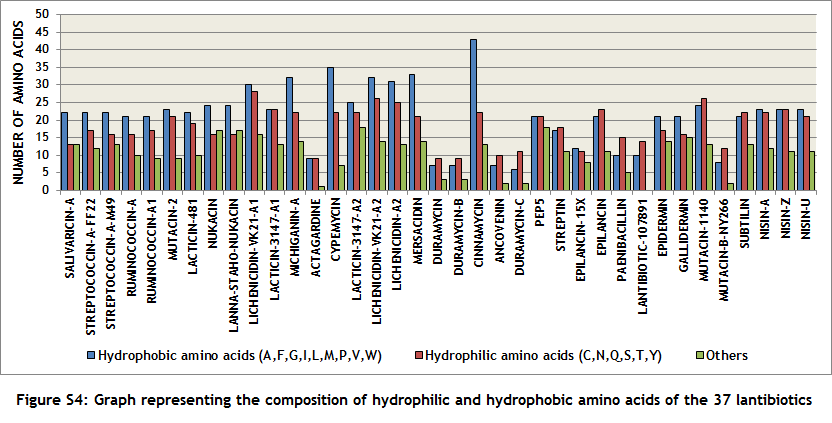
**

**Figure S4: Graph representing the composition of hydrophilic and hydrophobic aminoacids of the 37 lantibiotics**

**
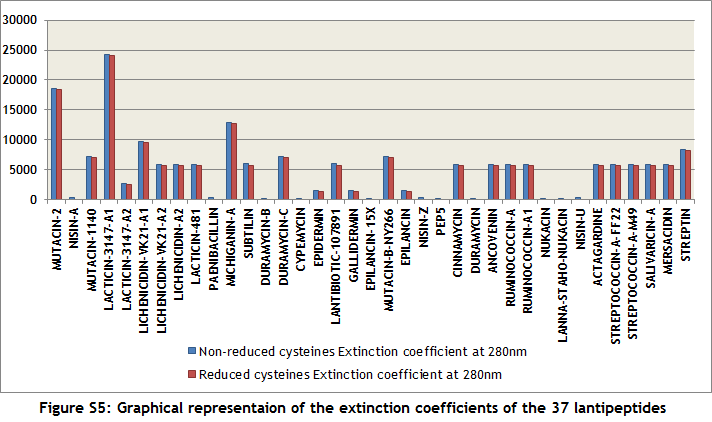
**

**Figure S5: Graphical representation of the extinction coefficients of the 37 lantipeptides**


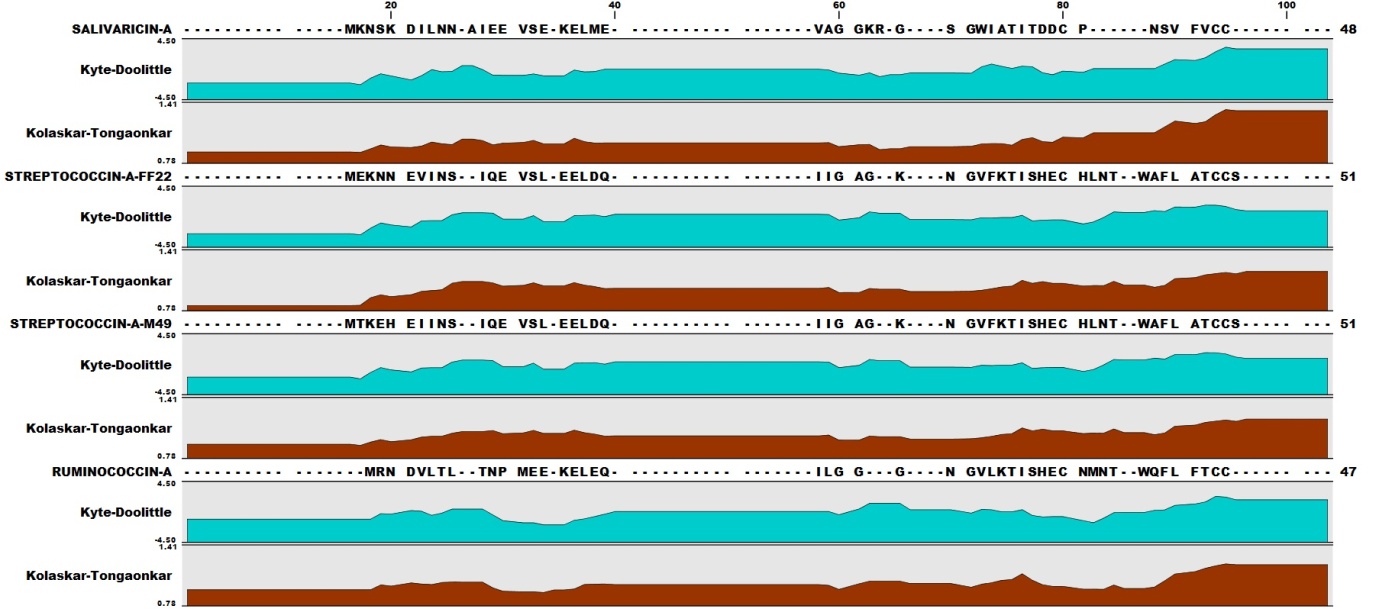

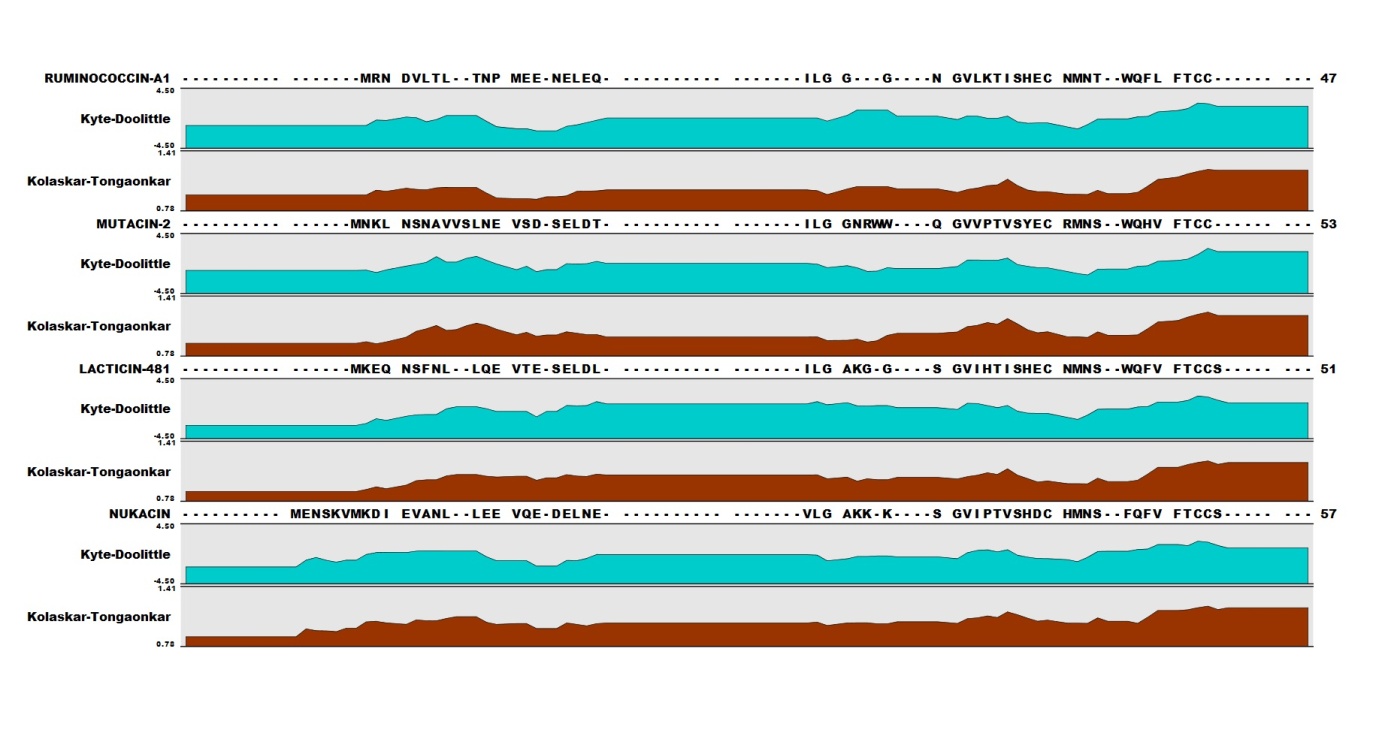

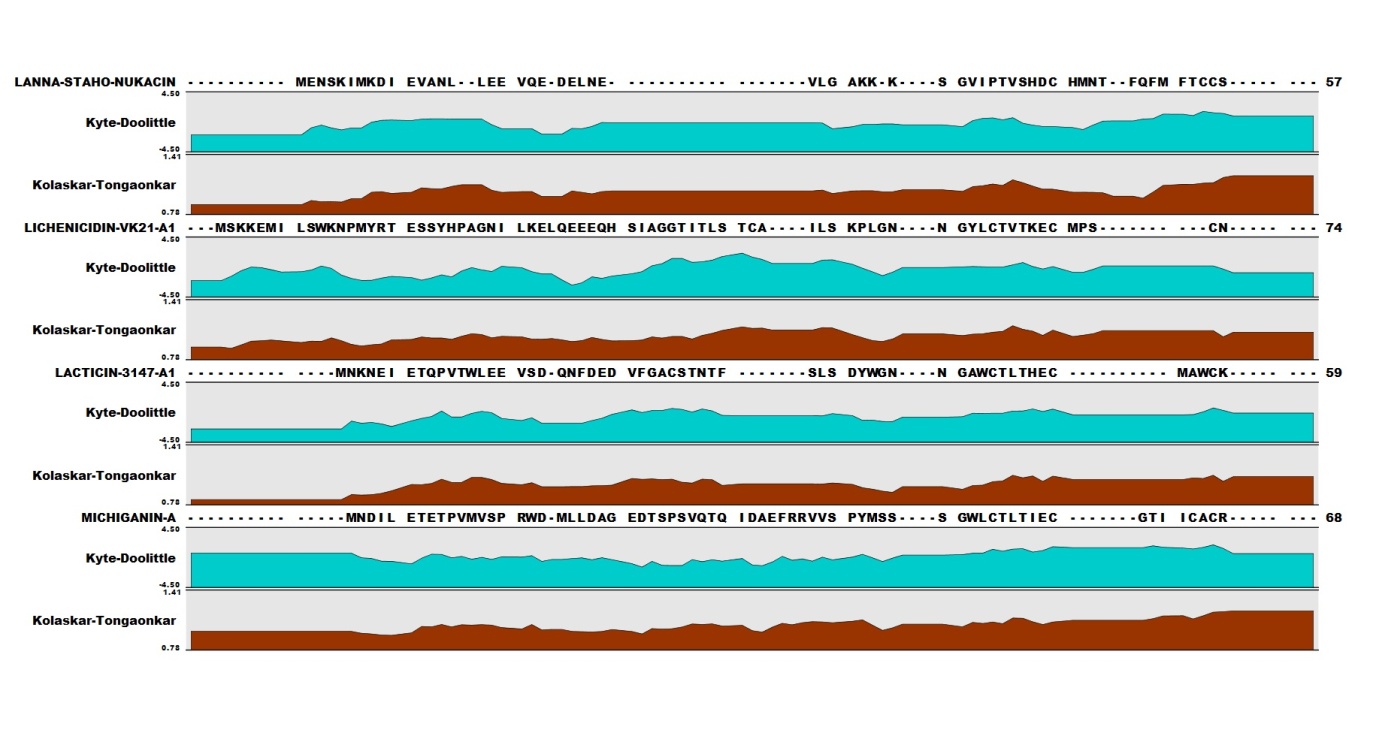


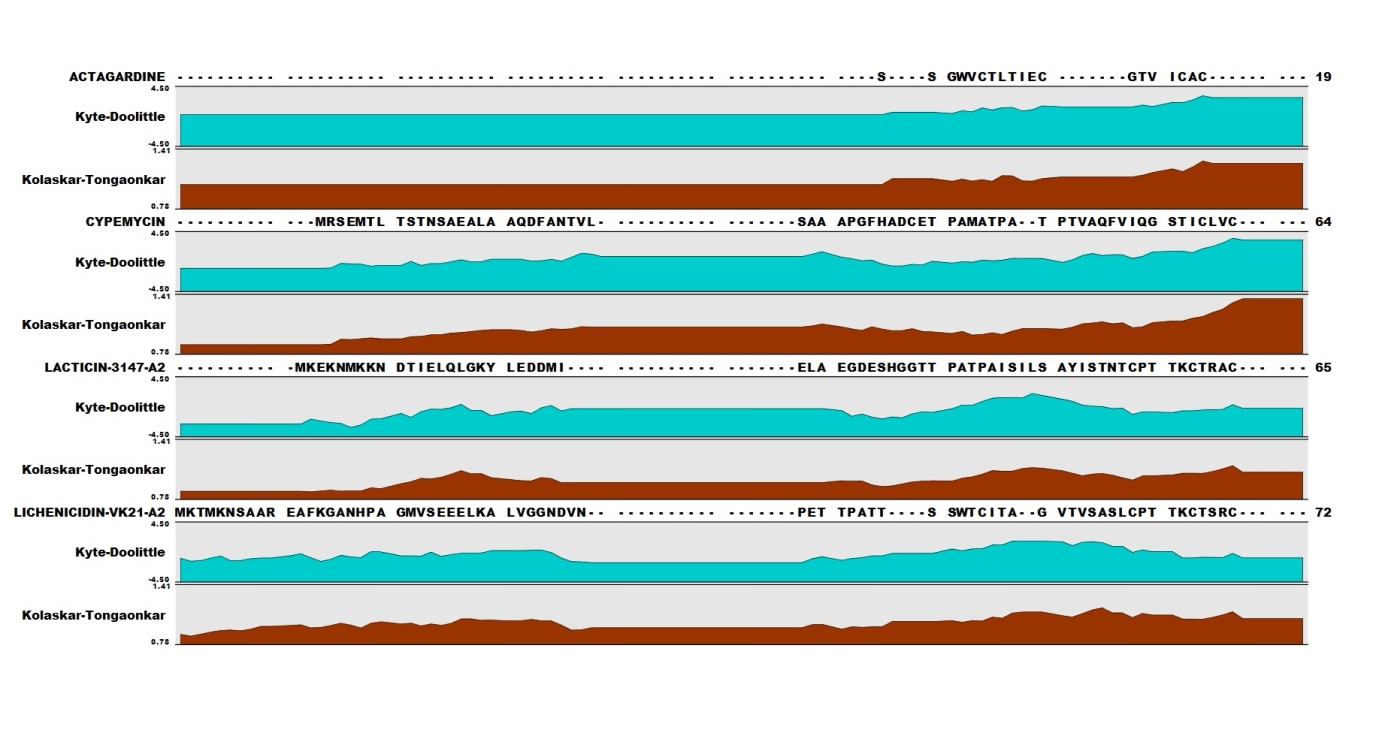

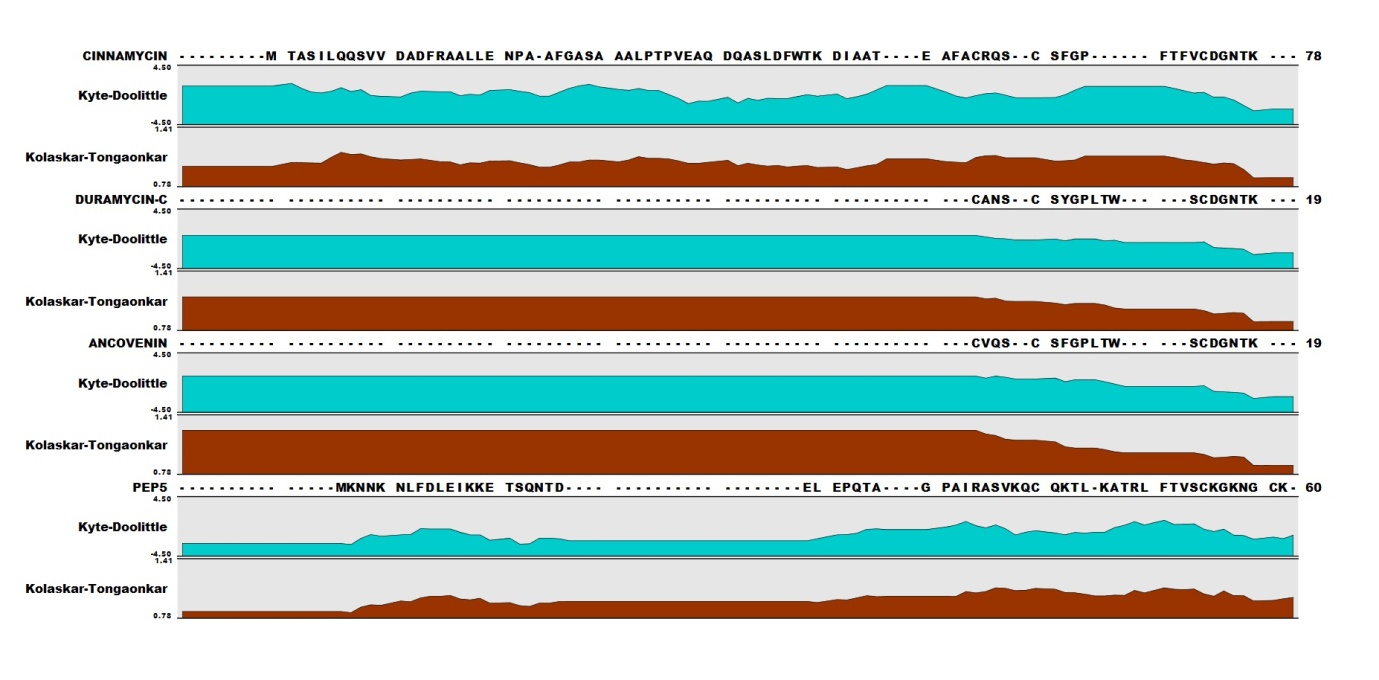

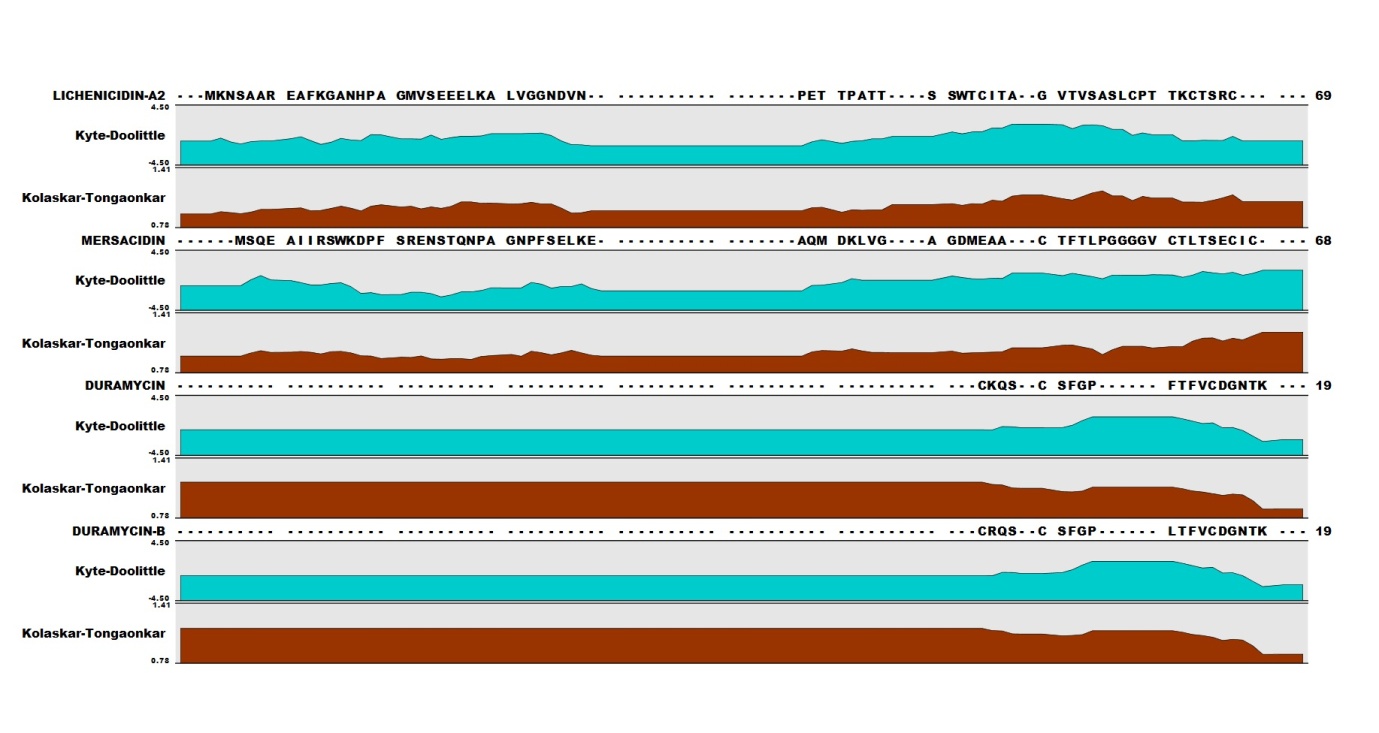


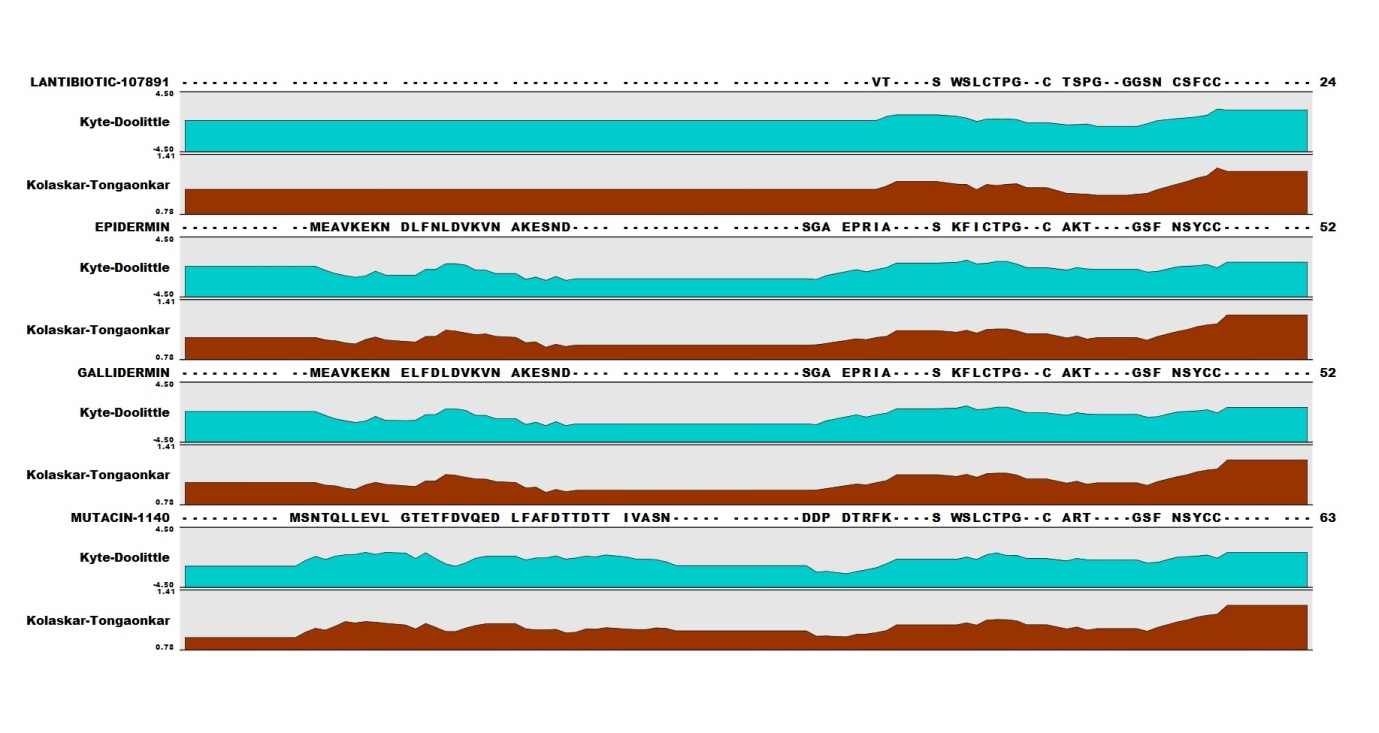

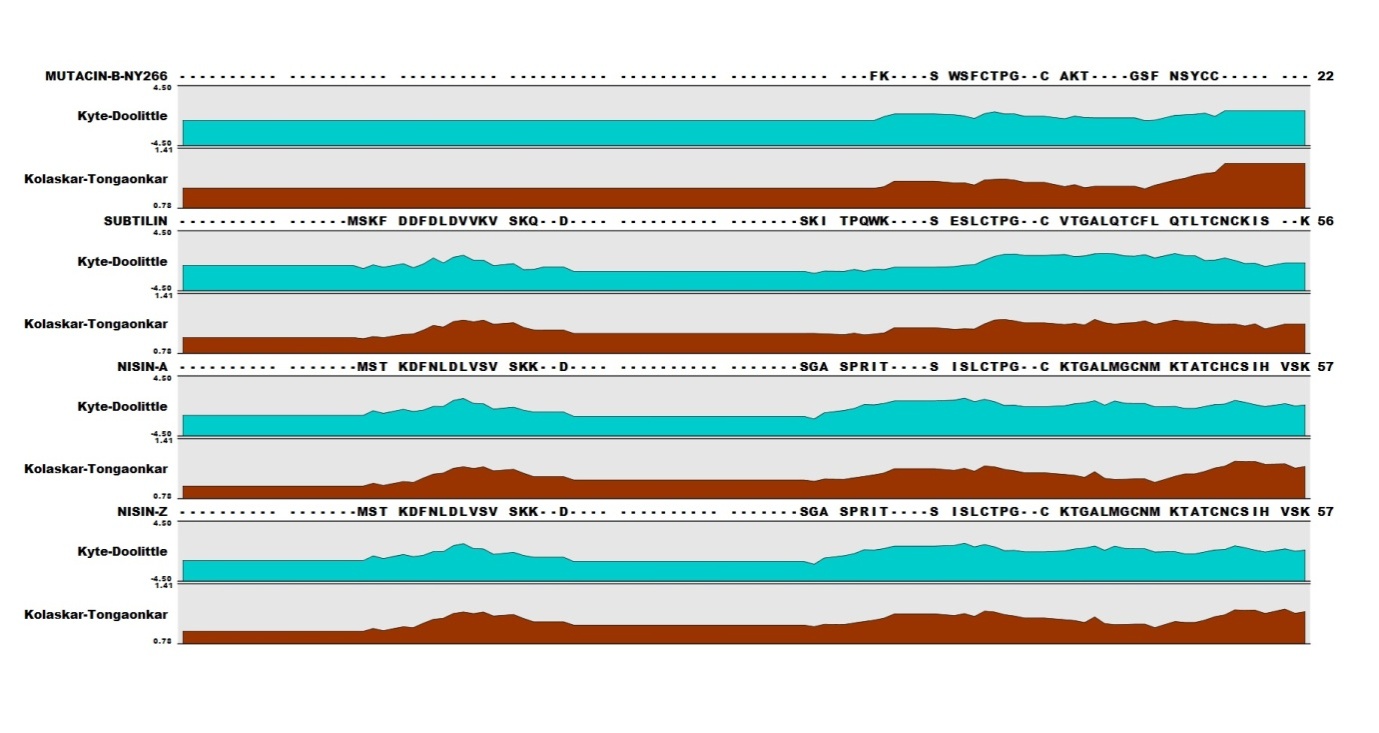

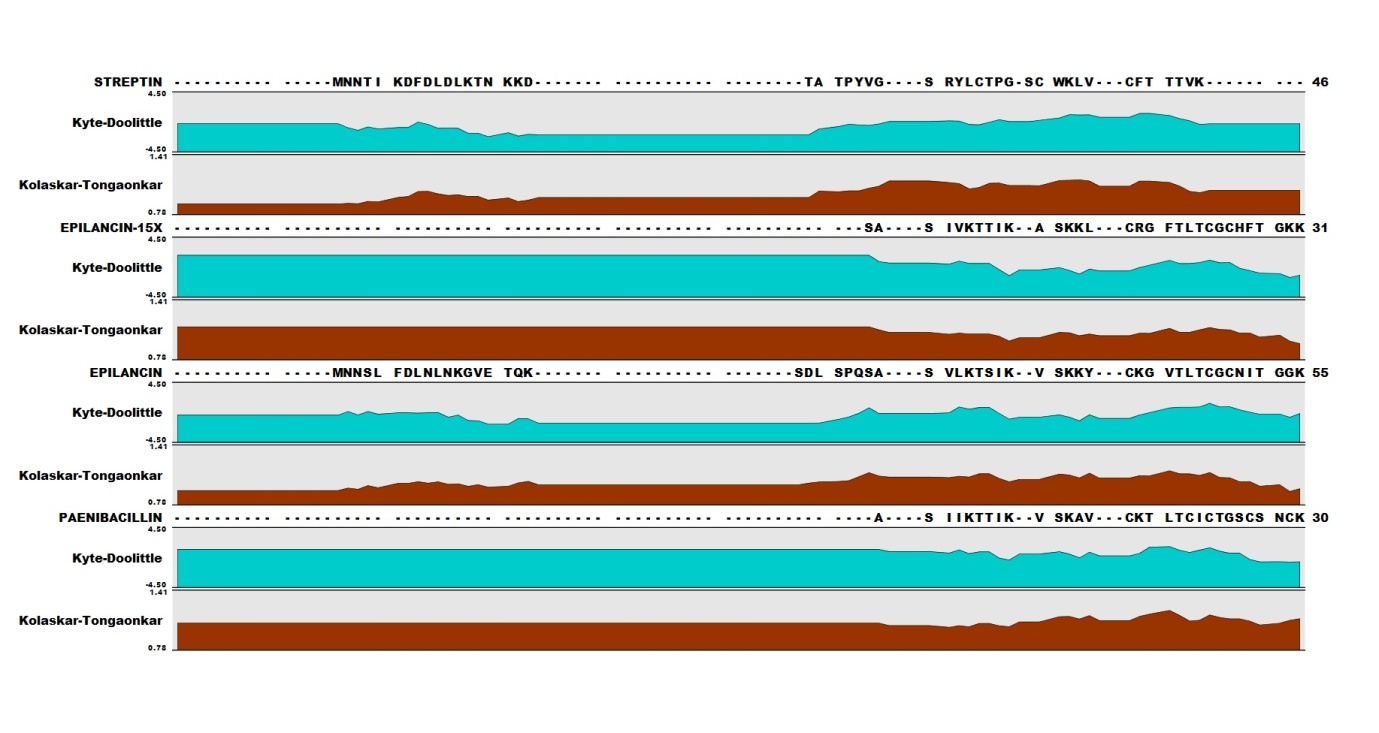


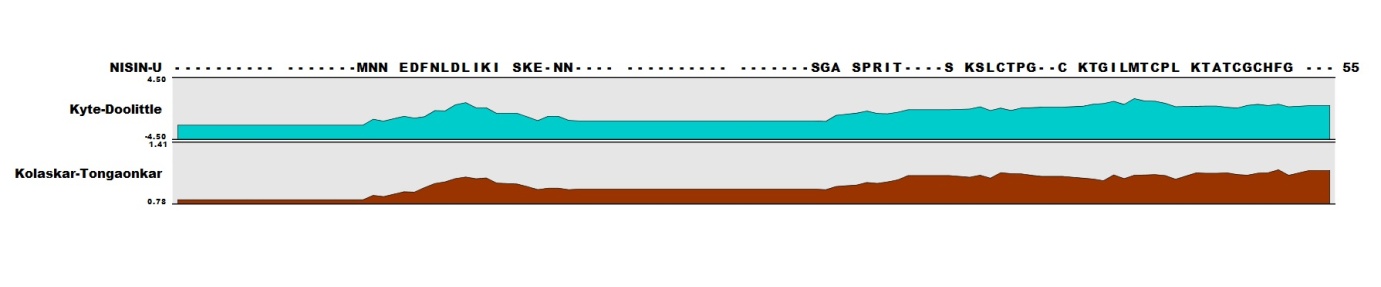


**Figure S6: Graphical representation of the hydropathy indices of the 37 lantipeptides. The area in sky-blue in the graph represent the hydropathy indices calculated by Kyte-Doolittle method, and the brown area in the graph represent the antigenic determinants of the lantibiotics, determined by semi-empirical method of Kolaskar and Tangaokar.**

**
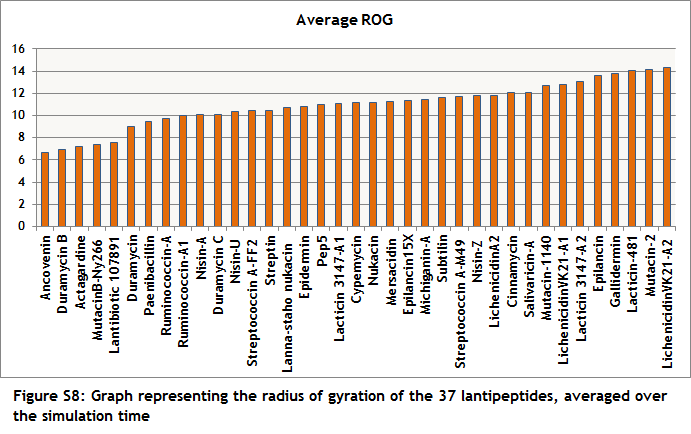
**

**Figure S7: Graph representing the radius of gyration of 37 lantibiotics, averaged over the simulation time**

**
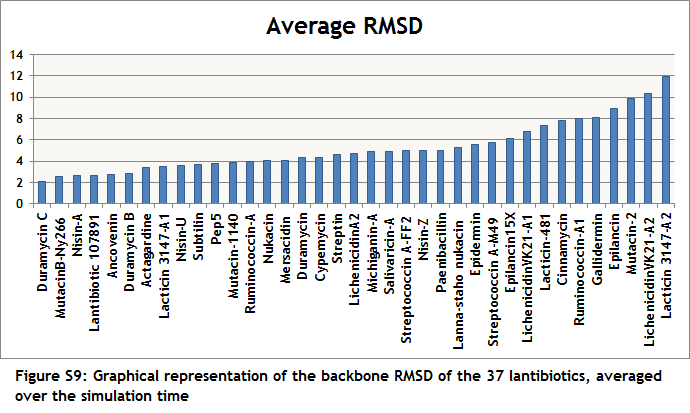
**

**Figure S8: Graphical representation of the backbone RMSD of the 37 lantibiotics , averaged over the simulation time**

**
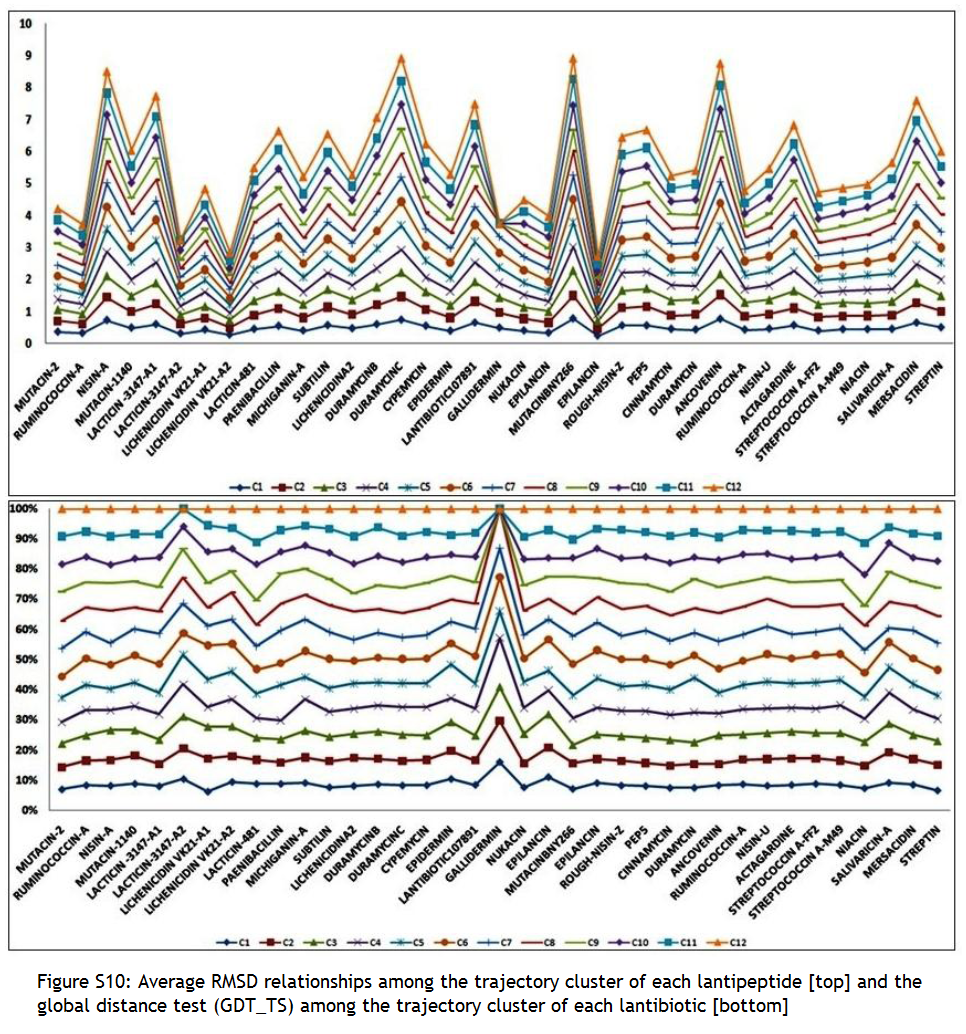
**

**Figure S9: Average RMSD relationships among the trajectory cluster of each lantipeptide [top] and the global distance test (GDT_TS) among the trajectory cluster of each lantibiotic [bottom]**

**Table S1: Lantibiotic sequences retrieved from UniProtKB**

| **Uniprot ID** | **Lantibiotic** | **Produced by** |
| --- | --- | --- |
| O54329 | Mutacin-2 | *Streptococcus mutans* |
| P83674 | Ruminococcin-A | *Ruminococcus gnavus* |
| P13068 | Nisin-A | *Lactococcus lactis* |
| O68586 | Mutacin-1140 | *Streptococcus mutans* |
| O87236 | Lacticin 3147 A1 | *Lactococcus lactis* |
| O87237 | Lacticin 3147 A2 | *Lactococcus lactis* |
| P86475 | Lichenicidin VK21 A1 | *Bacillus licheniformis* |
| P86476 | Lichenicidin VK21 A2 | *Bacillus licheniformis* |
| P36499 | Lacticin-481 | *Lactococcus lactis* |
| P86013 | Paenibacillin | *Paenibacillus polymyxa* |
| Q09T02 | Michiganin-A | *Clavibacter michiganensis subsp. michiganensis* |
| P10946 | Subtilin | *Bacillus subtilis* |
| P86720 | Lichenicidin A2 | *Bacillus licheniformis* |
| P36502 | Duramycin B | *Streptomyces* sp. |
| P36503 | Duramycin C | *Streptomyces griseoluteus* |
| E5KIB6 | Cypemycin | *Streptomyces* sp. |
| P08136 | Epidermin | *Staphylococcus epidermidis* |
| P85065 | Lantibiotic 107891 | *Microbispora*sp. |
| P21838 | Gallidermin | *Staphylococcus gallinarum* |
| Q9KWM4 | Nukacin | *Staphylococcus warneri* |
| P86047 | Epilancin 15x | *Staphylococcus epidermidis* |
| P80666 | Mutacin B-NY266 | *Streptococcus mutans* |
| Q57312 | Epilancin | *Staphylococcus epidermidis* |
| P29559 | Nisin-Z | *Lactococcus lactis* |
| P19578 | Pep5 | *Staphylococcus epidermidis* |
| P36501 | Streptococcin A-FF22 | *Streptococcus pyogenes* |
| B5MFD0 | Lanna-Stahonukacin | *Staphylococcus hominis* |
| Q2QBT0 | Nisin-U | *Streptococcus uberis* |
| Q54957 | Streptococcin A-M49 | *Streptococcus pyogenes*serotype M49 |
| P36500 | Salivaricin-A | *Streptococcus salivarius* |
| P36504 | Duramycin | *Streptomyces griseoverticillatus* |
| P29827 | Cinnamycin | *Streptomyces griseoverticillatus* |
| P83675 | Ruminococcin-A | *Blautiahansenii* |
| P43683 | Mersacidin | *Bacillus* sp. |
| P0C0H8 | Streptin | *Streptococcus pyogenes* |
| P56650 | Actagardine | *Actinoplanes liguriensis* |
| P38655 | Ancovenin | *Streptomyces* sp. |

**Table S2**: Validation of lantibiotic models

| **Lantibiotic** | **Prosa Z score** | **% of amino acids in Ramachandran regions (ProCheck)** | | | | **Verify 3D** | |
| --- | --- | --- | --- | --- | --- | --- | --- |
|  |  | **Most favoured regions** | **Additionally allowed regions** | **Generously allowed regions** | **Disallowed regions** | **Global quality score** | **Global quality Z-score** |
| Salivaricin A | -2.56 | 78.0 | 12.2 | 4.9 | 4.9 | 0.04 | 0.04 |
| Streptococcin A FF22 | -3.14 | 84.8 | 10.9 | 0.0 | 4.3 | 0.20 | -4.17 |
| Streptococcin A M49 | -3.35 | 87.0 | 4.3 | 2.2 | 6.5 | 0.18 | -4.49 |
| Ruminococcin A | -0.74 | 85 | 12.5 | 0 | 2.5 | 0.31 | -2.41 |
| Ruminococcin A1 | -3.52 | 60 | 20 | 5 | 15 | 0.18 | -4.49 |
| Mutacin 2 | -5.62 | 91.5 | 2.1 | 2.1 | 4.3 | 0.12 | -5.46 |
| Lacticin 481 | -3.56 | 91.1 | 6.7 | 0 | 2.2 | 0.05 | -6.58 |
| Nukacin | -0.50 | 82.7 | 11.5 | 3.8 | 1.9 | 0.29 | -2.73 |
| LannaStahoNukacin | -2.44 | 76.9 | 17.3 | 1.9 | 3.8 | 0.22 | -3.85 |
| Lichenicidin VK21 A1 | -2.07 | 85.7 | 14.3 | 0 | 0 | 0.20 | -4.17 |
| Lacticin 3147 A1 | -1.36 | 90.6 | 7.5 | 0 | 1.9 | 0.29 | -2.73 |
| Michiganin A | -2.32 | 78 | 11.9 | 3.4 | 6.8 | 0.31 | -2.41 |
| Actagardine | -8.35 | 93.3 | 6.7 | 0 | 0 | 0.18 | -4.49 |
| Cypemycin | -3.60 | 75 | 14.3 | 5.4 | 5.4 | 0.16 | -4.82 |
| Lacticin 3147 A2 | -2.11 | 67.9 | 21.4 | 3.6 | 7.1 | 0.36 | -1.61 |
| Lichenicidin VK21 A2 | -3.23 | 91.8 | 4.9 | 1.6 | 1.6 | 0.20 | -4.17 |
| Lichenicidin A2 | -1.03 | 79.3 | 12.1 | 1.7 | 6.9 | 0.24 | -3.53 |
| Mersacidin | -4.05 | 76.4 | 14.5 | 0.0 | 9.1 | 0.18 | -4.49 |
| Duramycin | -8.97 | 85.7 | 7.1 | 0 | 7.1 | -0.17 | -10.11 |
| Duramycin B | -5.33 | 85.7 | 14.3 | 0 | 0 | 0.06 | -6.42 |
| Cinnamycin | -2.69 | 84.1 | 11.6 | 0 | 4.3 | 0.34 | -1.93 |
| Ancoverin | -5.50 | 78.6 | 7.1 | 0 | 14.3 | 0.03 | -6.90 |
| Duramycin C | -4.34 | 82.2 | 11.1 | 4.4 | 2.2 | 0.11 | -5.62 |
| Pep 5 | -2.32 | 71.7 | 17 | 3.8 | 7.5 | 0.11 | -5.62 |
| Streptin | -3.76 | 80.0 | 17.5 | 2.5 | 0.0 | 0.29 | -2.73 |
| Epilancin 15X | -4.96 | 92.3 | 3.8 | 0 | 3.8 | 0.02 | -7.06 |
| Epilancin | -4.05 | 87.2 | 6.4 | 2.1 | 4.3 | 0.13 | 5.3 |
| Paenibacillin | -6.16 | 77.8 | 14.8 | 3.7 | 3.7 | -0.16 | -9.95 |
| Lantibiotic 107891 | -2.81 | 81.2 | 6.2 | 6.2 | 6.2 | 0.44 | -0.32 |
| Epidermin | -1.86 | 68.9 | 20 | 2.2 | 8.9 | 0.15 | -4.98 |
| Gallidermin | -0.95 | 77.8 | 11.1 | 2.2 | 8.9 | 0.32 | -2.25 |
| Mutacin 1140 | -3.64 | 82.1 | 12.5 | 1.8 | 3.6 | 0.21 | -4.01 |
| Mutacin BNY266 | -7.11 | 88.2 | 11.8 | 0 | 0 | -0.06 | -8.35 |
| Subtilin | -0.87 | 70.0 | 24.0 | 2.0 | 4.0 | 0.25 | -3.37 |
| Nisin A | -2.89 | 83.7 | 12.2 | 2 | 2 | 0.16 | -4.82 |
| Nisin Z | -3.06 | 75.5 | 16.3 | 4.1 | 4.1 | 0.19 | -4.33 |
| Nisin U | -1.78 | 78.3 | 19.6 | 0 | 2.2 | 0.41 | -0.80 |

**Table S3**: Prediction of disordered residues using DISOPRED3

| Lantibiotic | Protein-binding disordered residues | | Disordered region | |
| --- | --- | --- | --- | --- |
|  | Residues | Sequence | Residues | Sequence |
| 1 | - | - | 1-2 | MN |
| 2 | - | - | 1-2 | MR |
| 3 | - | - | 1-3 | MST |
| 4 | - | - | - | - |
| 5 | 1-6 | MNKNEI | 1-6 | MNKNEI |
| 6 | 1-11 | MKEKNMKKNDT | 1-11 | MKEKNMKKNDT |
| 7 | - | - | 1 | M |
| 8 | 46-48 | TSS | 1-3, 46-48 | MKT, TSS |
| 9 | - | - | 1 | M |
| 10 | - | - | - | - |
| 11 | - | - | 1-2 | MN |
| 12 | - | - | 1-2 | MS |
| 13 | - | - | 1-3 | MKN |
| 14 | - | - | 1 | C |
| 15 | - | - | 1-2 | CA |
| 16 | 1-9 | MRSEMTLTS | 1-9 | MRSEMTLTS |
| 17 | - | - | 1-2 | ME |
| 18 | - | - | - | - |
| 19 | - | - | 1-2 | ME |
| 20 | 1 | M | 1-4 | MENS |
| 21 | - | - | 1, 31 | S, K |
| 22 | - | - | - | - |
| 23 | 1-2 | MN | 1-3 | MNN |
| 24 | 1-3 | MST | 1-3 | MST |
| 25 | 1-2 | MK | 1-4 | MKNN |
| 26 | - | - | 1 | M |
| 27 | - | - | 1 | C |
| 28 | - | - | - | - |
| 29 | 1-2 | MR | 1-2 | MR |
| 30 | - | - | 1-2 | MN |
| 31 | - | - | - | - |
| 32 | - | - | 1-2 | ME |
| 33 | - | - | 1 | M |
| 34 | 1-2 | ME | 1-4 | MENS |
| 35 | 1 | M | 1-3 | MKN |
| 36 | - | - | 1 | M |
| 37 | - | - | 1-2 | MN |
